# Advanced real-time recordings of neuronal activity with tailored patch pipettes, diamond multi-electrode arrays and electrochromic voltage-sensitive dyes

**DOI:** 10.1007/s00424-020-02472-4

**Published:** 2020-10-13

**Authors:** Bernd Kuhn, Federico Picollo, Valentina Carabelli, Giorgio Rispoli

**Affiliations:** 1 Optical Neuroimaging Unit, OIST Graduate University, 1919-1 Tancha, Onna-son, Okinawa, Japan; 2 Department of Physics, NIS Interdepartmental Centre, University of Torino and Italian Institute of Nuclear Physics, via Giuria 1, 10125 Torino, Italy; 3 Department of Drug and Science Technology, NIS Interdepartmental Centre, University of Torino, Corso Raffaello 30, 10125 Torino, Italy; 4 Department of Biomedical and Specialist Surgical Sciences, University of Ferrara, Via Luigi Borsari 46, 44121 Ferrara, Italy

**Keywords:** Patch clamp, Fast cellular perfusion, Multi-electrode recording, Diamond sensors, Voltage-sensitive dyes, Two-photon microscopy

## Abstract

To understand the working principles of the nervous system is key to figure out its electrical activity and how this activity spreads along the neuronal network. It is therefore crucial to develop advanced techniques aimed to record in real time the electrical activity, from compartments of single neurons to populations of neurons, to understand how higher functions emerge from coordinated activity. To record from single neurons, a technique will be presented to fabricate patch pipettes able to seal on any membrane with a single glass type and whose shanks can be widened as desired. This dramatically reduces access resistance during whole-cell recording allowing fast intracellular and, if required, extracellular perfusion. To simultaneously record from many neurons, biocompatible probes will be described employing multi-electrodes made with novel technologies, based on diamond substrates. These probes also allow to synchronously record exocytosis and neuronal excitability and to stimulate neurons. Finally, to achieve even higher spatial resolution, it will be shown how voltage imaging, employing fast voltage-sensitive dyes and two-photon microscopy, is able to sample voltage oscillations in the brain spatially resolved and voltage changes in dendrites of single neurons at millisecond and micrometre resolution in awake animals.

## Introduction

One of the outstanding features of the nervous system is the electrical activity of its neurons and how this activity spreads along the neuronal network. The recording of this activity allows, on the one hand, the detailed study of the mechanisms generating and modulating it, and on the other hand, to have clues on how the coordinated activity of neuronal populations generates internal brain states and behaviour. This paper reviews some recent and promising innovations of techniques able to record the real-time neuronal activity, from the synaptic transmission to single cells to brain slices to in vivo mammalian preparations, all with maximal biocompatibility. This review focuses on improved patch techniques, extracellular multi-electrode systems, the detection of oxidizable neurotransmitter release and voltage imaging.

## Advanced patch-clamp recordings

Patch-clamp recording, originally developed in the late 1970s for measuring single ion channel currents, rapidly became the gold standard for the measurement of cellular electrical activity with high temporal resolution and precision [41, 89]. This prompted an exceptional knowledge advancement in almost every biological field such as the structure-function relationship of membrane proteins, cell signalling, hormone and neurotransmitter secretion, nuclear membrane trafficking and neuronal computation. These advancements have been possible because of several improvements to the original technique. This chapter presents upgrades aimed to optimize seal gaining, electrical recording and intra- and extracellular perfusion, specifically for cells mechanically dissociated or embedded in a tissue sample.

### Reliably attaining high seal resistance

Every patch-clamp recording configuration (cell attached, excised patch or whole cell) requires to consistently attain a tight seal between the cell membrane and the pipette glass. The day-to-day variability in attaining a sufficient seal resistance (*R*_s_) has been a challenge for generations of scientists [69] and has given rise to all sorts of laboratory tales about how to prepare pipettes, solutions and cells. Although this technique has been widely used for about 50 years, the molecular nature of the membrane-glass interaction has not been fully clarified yet [100, 102]: however, decades of experience have brought some firm conclusions.

**Fig. 1:**
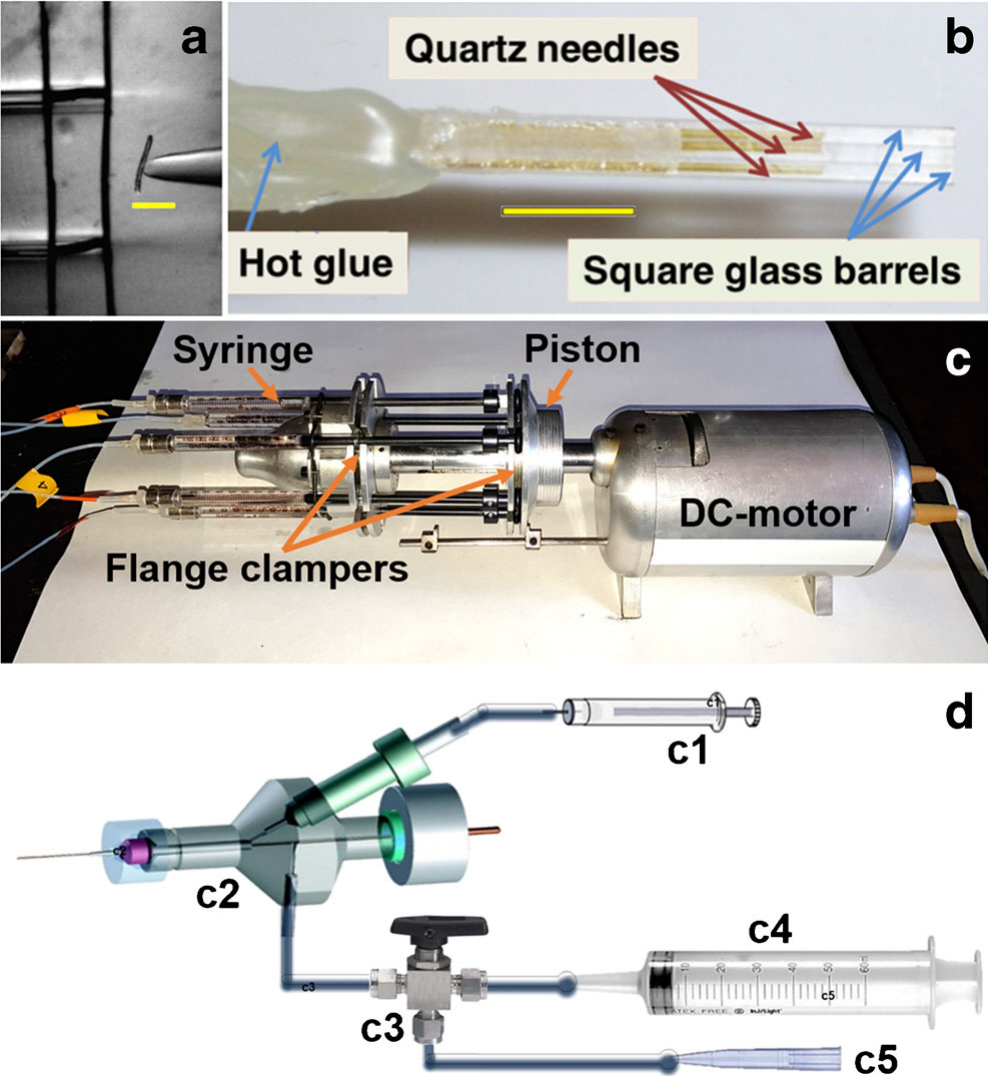
Setup for fast extracellular and intracellular perfusion. **a** Isolated skeletal muscle cell in perfusion (yellow scale bar is 200 μm); the boundary separating two adjacent streams is visible above and below the cell. **b** Assemblage of a multi-barrelled perfusion pipette (yellow scale bar is 5 mm), whose tip is shown enlarged in the microscope image in **a**. **c** Servocontrolled DC motor coupled to the piston of five precision syringes, whose plunger flange and barrel flange are held in place all at once by two cylindrical clampers; this system can accommodate up to six syringes (coupled to six perfusion lines connected to a six-barrelled pipette). **d** Representation of the setup for cytosolic perfusion: by moving the piston of a precision syringe (c1) with a micromanipulator (not shown), a positive pressure is applied to the perfusion tube (containing the test solution to be injected into the cytosol), inserted in the pipette lumen via a custom side port drilled in the holder (c2). The standard side port is connected, via a three-way valve (c3), to a 50-ml syringe (c4) to apply strong positive pressure to clean the pipette and the cells or to a mouthpiece (c5) or a syringe, to attain the seal (modified from [7, 8, 71, 92, 113])

First, in our experience, the variability in attaining the seal is entirely due to the cell quality (too soiled with connective tissue, extracellular matrices, or tissue debris, or too fragile to sustain the sealing process) and not to the pipette, if it is clean and fire polished. It is therefore necessary to carefully keep everything free of dirt that could contaminate the pipette glass, from the capillary to the finalized pipette, as well as the microscope stage, the puller, the pipette storage jar, the platinum filament used to fire polish the pipette tip and the pipette holder. To avoid metal evaporation onto the pipette tip, it is better to uniformly coat the filament by dipping it in glass powder (made from capillaries fine ground with pestle and mortar after removing all the ink markings) when heated to yellow colour. Before pulling, the capillaries must be thoroughly cleaned with ethanol, avoiding touching them with bare hand especially in their centre, i.e. where they will be thinned to form the two pipette tips. Fire polishing improves the sealing capacity of the pipette, but not because it removes any contaminants left on the tip, for instance after coating with insulating agent, as proposed by many authors [74, 90]. Indeed, in our experience, fire polishing improves the sealing capacity of non-coated pipettes as well, while it negligibly affects one of soiled pipettes. The fire polishing rather smooths the surface of the pipette tip, that can be rough at the molecular level due to the abrupt separation from its companion during pulling [64], and thickens its wall. It is therefore likely that the fire-polished pipette tip can seal a larger portion of membrane with respect to the one sealed by a non-polished tip, increasing *R*_s_. Holding negative voltage or negative pressure inside the pipette accelerates the sealing process, due to a voltage-dependent (possibly arising from electroosmosis [100]) or pressure-dependent creep of the cell membrane into the pipette tip, increasing therefore the membrane surface sealed on the glass. Although the surface of the sealed membrane is much smaller than the cell area, it anyway sticks the cell to the pipette so firmly, that even extremely elongated cells can be extracellularly perfused (Fig. 1a, b and c) without the perfusion stream tearing them off the patch pipette [71]. This mechanical stability is also demonstrated by the following observation: when a large bleb is formed inside the pipette after applying a too large depression inside the pipette, it is possible to expel most of the bleb by applying positive pressure, without losing the seal. This indicates that the membrane sealed to the glass form lumps so immovable that the non-sealed lipids could flow around them quite freely. Fire polishing is crucial to gain a seal on cells in a tissue sample or slice, or freshly dissociated from tissue with mechanical manipulations. Unpolished pipettes are anyway able to seal on stable cell lines or on enzymatically dissociated cells, but they consistently give smaller *R*_s_ with respect to the polished ones.

Second, it is not a general rule that different types of glasswork better on different cell types [74]: in our experience, the borosilicate glass (50- or 100-μl microcapillary tubes) is able to attain tight seals on a wide variety of cells, being mechanically or enzymatically dissociated from tissue, still inserted in it, or in culture, and even on giant unilamellar vesicles (GUV) of many different lipid compositions [6, 11]. The critical parameter is instead the pipette tip diameter: as a rule of thumb, it should be less than one-fifth of the cell or GUV diameter since their basic shapes are spheres and cylinders. Larger pipettes will not seal, will destroy the membrane or entirely suck the cell inside the pipette; smaller ones seal better but they will give too high access resistance (*R*_a_) in whole-cell configuration. Consistent tight seals with borosilicate glass microcapillaries were obtained on sensory cells (from reptilian, amphibian and fish retinae [6, 11]), neurons (horizontal cells, bipolar cells and ganglion cells from vertebrate retinae), culture cells (HEK 293, NB4, MDA-MB-231, COS, HeLa, A549 and COS-7), muscle cells (cardiac myocytes and muscle fibres of mice), erythrocytes and some white cells (from amphibian, reptilian, fish and human blood) and GUVs prepared by electroformation using different combinations (from one to up to four) of the following lipids: egg phosphatidylcholine, phosphatidylglycerol, cholesterol, dipalmitoylphosphatidylcholine, nitrobenzoxadiazol-labelled phosphatidylethanolamine and dansyl-labelled phosphatidylethanolamine. Glasses with low softening temperatures (as the ones having high lead content) are not as “universal sealers” as the borosilicate glass, which has the additional advantage of producing low noise, important for single-channel recordings. Noise can be further reduced with elastomer coating [90].

Third, when searching for a suitable cell, the pipette must be kept outside the bath, and, once in the bath, it is necessary to quickly head to the cell while keeping a constant positive pressure in the pipette (Fig. 1d), so the ejected pipette solution blows away any organic debris from the membrane target and the pipette tip. This “pressure cleaning” is not able to remove connective tissue or other organic material strongly sticking to the membrane of the targeted cell; in this case, specific proteolytic enzymes can be applied. Moreover, only with fire-polished pipettes, it is sometimes possible to gain a seal with the same pipette more than once, especially with stable cell lines (if the culture medium was carefully substituted with an extracellular solution) or cells freshly dissociated from tissue that underwent enzymatic treatment (if a quick progression from cell to cell prevents soiling of the pipette). Although *R*_s_ is constantly decreasing from one trial to the other, in our experience, it is possible to have *R*_s_ > 1 GΩ to the fourth consecutive seal with the same pipette.

Finally, proteins inserted in the pipette solution prevent seal formation, so they must be applied with a pipette perfusion system (Fig. 1d), or by filling the pipette tip with a solution lacking the protein, and backfilling the pipette with the same solution containing the protein. Contrary to many laboratory tales, the lack or the presence of Ca^2+^ do not promote or prevent the seal formation [85]: the sealing process with borosilicate glass is not significantly affected by concentrations up to 30 mM BAPTA or 30 mM Ca^2+^ in the pipette solution [91, 93].

### Pressure-polished pipette

The whole-cell configuration is the most widely used patch-clamp recording technique. The major limitation of this technique is the patch pipette geometry: its tapered shank and its small tip opening give high *R*_a_ and constitute the dominant barrier to molecular diffusion between the pipette and cell cytosol [86]. This shortcoming

1. gives errors in membrane potential control in the presence of large currents, due to the voltage drop across the *R*_a_ and produces intracellular ion accumulation or depletion, depending upon the current direction (reducing therefore the electrochemical gradient of the permeating ions);
2. impedes the precise measure of current onset and offset kinetics in the presence of large cells (i.e. having large electrical capacitance), due to the often too large time constant of charging the cell membrane capacitance through the *R*_a_;
3. slows down the rate of exogenous molecules incorporation via the patch pipette.

**Fig. 2:**
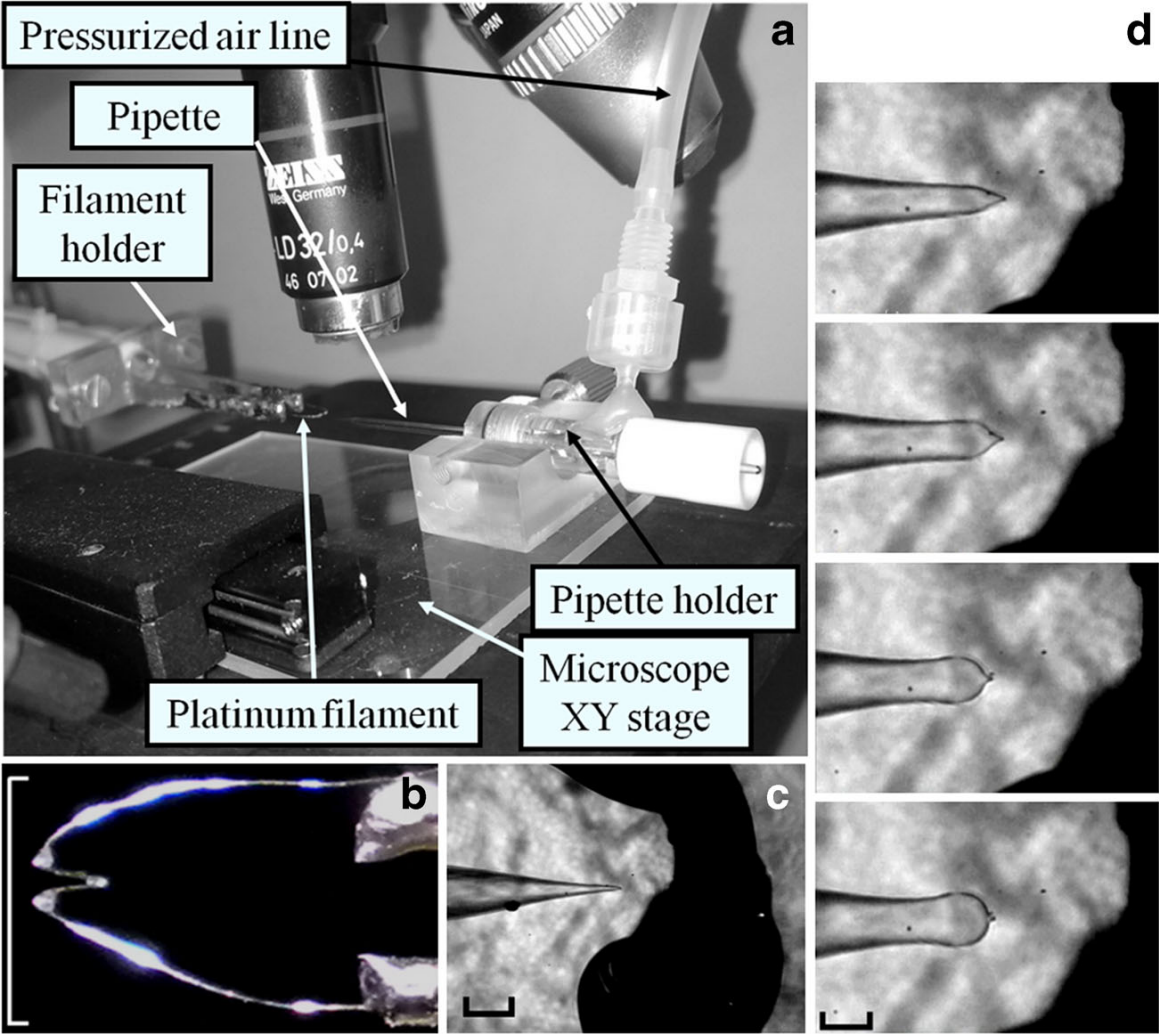
Fabrication of pressure-polished patch pipettes. **a** View of the setup’s main elements: the upright microscope, the microscope stage carrying the pipette holder connected to the pressurized line and the platinum filament. **b** The “omega” shaped, glass-coated platinum filament tin soldered to the holder; white scale bar: 5 mm. **c** A pipette before pressure polishing, aligned in the central bend of the filament, to ensure uniform heating; black scale bar: 40 μm. **d** Progressively enlarged pipette shanks obtained with progressively longer heating duration (approximately, from top to bottom: 1.9, 2.4, 3.0 and 3.5 s), while keeping the pressure inside the pipette lumen constant. (modified from [6, 11])

**Fig. 3:**
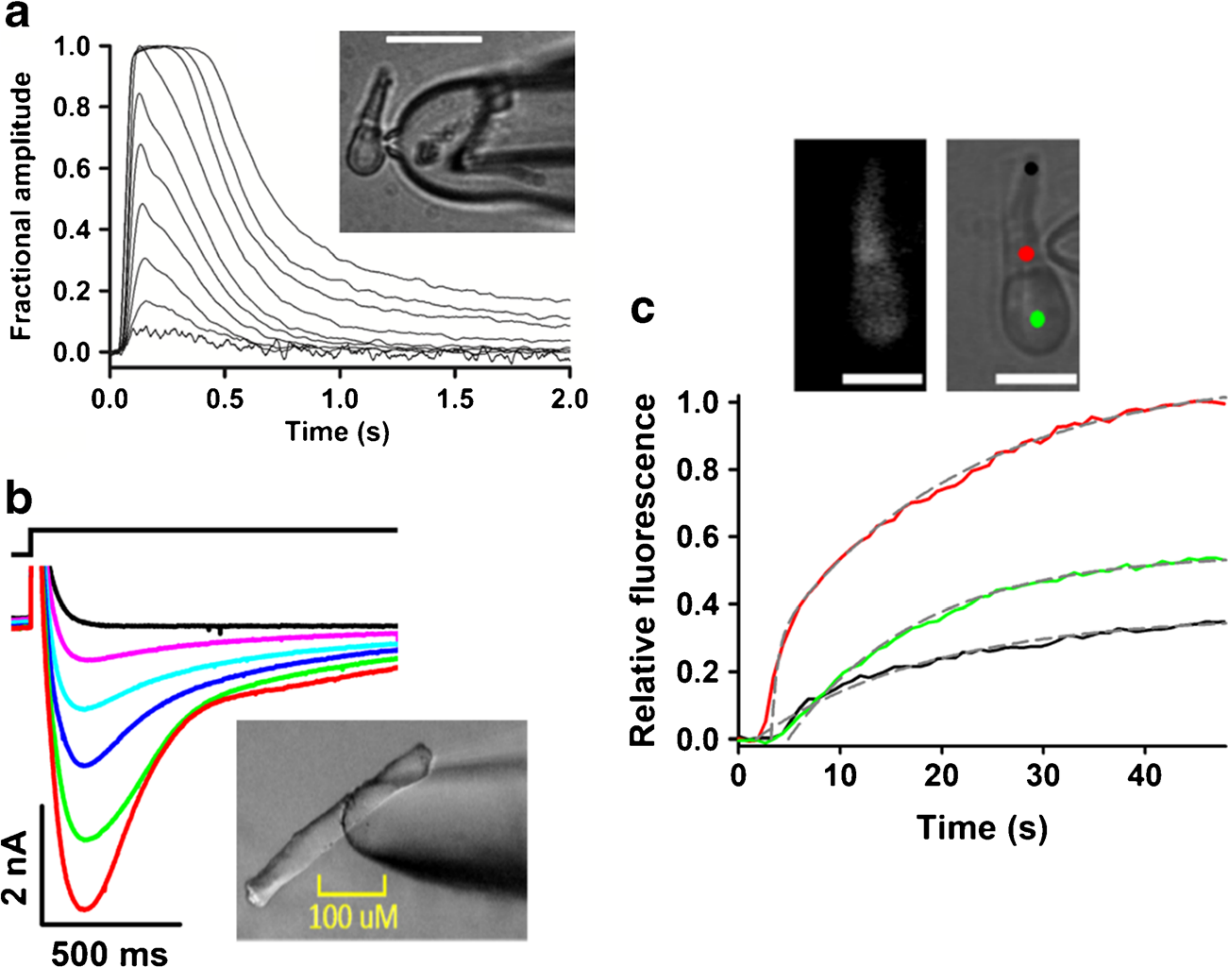
Application examples of pressure-polished pipettes. **a** Fractional suppression of light-sensitive current (5–9 responses averaged, average current amplitude: 21.3 pA) to 1 ms flashes delivering 1.14 × 10^2^, 5.70 × 10^2^, 1.14 × 10^3^, 2.32 × 10^3^, 4.49 × 10^3^, 8.68 × 10^3^, 1.77 × 10^4^, 9.55 × 10^4^, 1.85 × 10^5^ and 3.76 × 10^5^ photons/μm^2^ recorded from a zebrafish cone (with standard internal and external solutions [7]) by means of a pressure-polished pipette. A pulled quartz tube is inserted as close as possible to its tip (inset). **b** Ca^2+^ current waveform recorded from a murine muscle cell of flexor digitorum brevis (inset) at various voltages (black: − 60 mV, pink: − 10 mV, cyan: 0 mV, blue: + 30 mV, green: + 10 mV, red: + 20 mV; black trace on top is voltage timing). Ca^2+^ current is obtained by subtracting the current at various voltages in a stream of the extracellular solution and the current recorded at the same voltages in an adjacent stream containing 50 μM nifedipine (a Ca^2+^ channel blocker). Internal and external solutions are designed to block the Na^+^ and K^+^ currents [71]. **c** A zebrafish retinal cone (inset, left panel; scale bar: 10 μm; *R*_a_ ~ 4.1 MΩ) is subject to injection of Lucifer yellow (40 μM) starting at time 0. The cell fluorescence intensity vs time, integrated with the black, red and green regions of inset, right panel (showing the fluorescence after 50 s of continuous injection) normalized to the maximal intensity recorded in the site of dye injection (red region), is shown with the same colour of the corresponding region. The experimental curves can be fitted (dotted grey lines) with a biexponential equation (time constants fitting the red curve: 0.6 s and 19 s) and with a monoexponential one (green curve: 13 s; black curve, 17.5 s) (modified from [7])

These problems can be circumvented, all at once, by widening the patch pipette shank independently of the tip opening diameter (Fig. 2) through the calibrated application of heat (applied uniformly outside of the shank with an omega-shaped incandescent filament) and air pressure (applied to its lumen, filtered to 0.2 μm), with a custom-made inexpensive setup (Fig. 2a [6, 11, 48]). This “pressure polishing” method is just an application of the blowing glass technique, invented more than 2000 years ago in Rome, during the Augustan age, and brought to perfection in Murano Island, Venice, since thirteenth century AD. Given the “sealing power” of the borosilicate glass microcapillaries, the pressure polishing method has been optimized just for this glass type. The pipette shank geometry and its tip opening diameter can be precisely controlled by adjusting: (1) the current intensity passing through the filament, (2) the relative position of the pipette in respect to the filament, (3) the duration of this current flow and (4) the pressure intensity maintained while the current is flowing in the filament (i.e. while the pipette glass is softened). The most efficient strategy is to optimize the parameters (1) (for a filament as in Fig. 2b, the current is 1.2 A), (2) (Fig. 2c shows this positioning) and (4) (air pressure held constant to 4 atm), and keep them fixed throughout the pressure polishing process, while parameter (3) is left to be adjusted each time in order to obtain the desired shank profile and tip opening diameter. Progressively longer heating applications (while keeping a constant pressure) applied to a pulled pipette (Fig. 2c) produce larger and larger pipette shanks (Fig. 2d). The pipette tip opening can be adjusted independently by the shank size: if the tip is too large, the fire polishing procedure is repeated in the absence of pressure until it is reduced to the desired size; if it is too small, then the pressure polish procedure is repeated on a pipette pulled with a larger tip. Pipettes with the same tip opening but with progressively enlarged shank have a progressively smaller resistance (*R*_p_) and therefore smaller *R*_a_. The *R*_a_ yield by a pipette with the widest shank (Fig. 2d, bottom) is about a quarter of the *R*_a_ of a conventional pipette (Fig. 2c). The increased *R*_a_ allows to keep the tip opening relatively small, promoting the seal on small and delicate cells, improving the seal on ordinary ones, minimizing the pipette washout and being able to reliably collect very large currents from huge cells. Indeed, it is possible to consistently record with pressure-polished pipette current as small as a few picoamperes (Fig. 3a) from tiny and fragile cells as zebrafish cones (10 μm long and 2 μm largest diameter, insets of Fig. 3a and c; typical *R*_p_ ~ 3 MΩ, yielding *R*_a_ ≤ 7 MΩ; [7]). The small tip opening of these pipettes (without compromising *R*_a_) reduces the pipette washout of cell soluble proteins to a point that is possible to record from zebrafish cones for long times (> 20 min), without any significant change in their light sensitivity, photoresponse waveform, light adaptation and dark current amplitude [7]. At the same time, the pressure-polished pipette allows reliable recordings from large and robust cells as isolated skeletal muscle fibres (40-μm diameter, 300 μm length, Fig. 3b inset; [71]) with currents of several nanoamperes (Fig. 3b; typical *R*_p_ ~ 200 kΩ, yielding *R*_a_ as low as 500 kΩ and no more than 3-mV voltage error for a 6-nA peak current).

### Extracellular perfusion

In many experiments, it is necessary to quickly apply and remove molecules (as channel blockers, neurotransmitters, ions, etc.) to the cell’s external side. Given the mechanical stability of the strong seals obtained with the pressure-polished pipettes, it is possible to perfuse the patch-clamp recorded cell with different solutions pouring out of a multi-barrelled pipette that is moved horizontally in front of the cell (Fig. 1a). A linear flow is obtained with square glass pipettes when the stream speed is 15 μl/min that gives also sharp boundaries between perfusion streams, avoiding at the same time that the perfusion stream tears the cell off the patch pipette. When the perfusion pipette is moved on a horizontal plane (by hand or by a computer-controlled step motor), the solution change occurs (in ~ 50 ms) when the cell crosses the sharp boundary separating two adjacent streams (visible in Fig. 1a) [71, 92, 113]. Convenient design is to use the (commercially available) three-barrel square glass capillaries (500 μm of side) that can be glued together to scale up the number of perfusion lines. Each solution is fed to the corresponding barrel through the Luer connector of a quartz needle glued inside it (Fig. 1b). All the perfusion lines should be built by using tubes and quartz needles of the same length and diameter, in order to have the same hydraulic resistance in each perfusion line. This is particularly important in the case of gravity-fed solutions, because a solution flowing slower than the one flowing in an adjacent barrel could be contaminated by the latter. For this reason, it has opted for a perfusion system employing precision syringes whose piston is moved by a single servocontrolled DC motor (to avoid electrical noise, Fig. 1c), which forces all solutions to flow at the same rate [92, 113].

### Intracellular perfusion

Pressure-polished pipettes increase the rate of molecular diffusion between the pipette and the cell interior, allowing to perfuse even large molecules and to modulate a multitude of cellular processes, such as signal transduction cascades, in real time [8]. However, proteogenic molecules stick to the glass tip and thereby prevent seal formation. Therefore, it is important to add them to the pipette solution after the seal is attained. Moreover, in some experiments, it is important not to apply a particular molecule to the cytosol at the beginning of the whole-cell recording but at a desired later time point. Besides improving the electrical recordings, the pressure-polished pipettes could accommodate pulled perfusion capillaries fabricated with quartz or plastic (such as polyethylene or polypropylene) tubes that can be easily softened with a Bunsen flame. The pulled end of these tubes can be positioned very close to the pipette tip, i.e. very close to the cell cytosol, when inserted in the enlarged shanks (Fig. 3a, inset).

The solution containing the molecule under test can be ejected by applying pressure to the tube lumen, resulting in the intracellular application of even large molecules such as proteins or antibodies [7, 8, 11]. The pressure can be simply applied by commercially available pressure generators or by a micromanipulator coupled to a piston of a 10-μl precision syringe (Fig. 1d). In the latter case, it is necessary to electrically insulate the piston from the micromanipulator with Teflon, and grounding the micromanipulator, to avoid introducing noise in the recording. The molecule incorporation occurs typically in less than 1 min from the pressure onset instead of several minutes in the case of conventional pipettes. This is even the case in one of the most unfavourable cell types, the zebrafish cone (Fig. 3a, c). Despite the small pipette tip diameter, necessary to record from this tiny and fragile cells, and the presence of a very packed disk stack that hinder lateral molecular diffusion, full loading of the fluorescent dye during whole-cell experiments occurs within 1 min (Fig. 3c).

## Detection of neurotransmitter release and action potentials by diamond-based MEAs

The patch-clamp technique is of paramount importance in the study of the mechanisms generating and modulating single-cell electrical activity. But despite 50 years of continuous improvements, including the ones presented here, it has still many drawbacks. It is indeed time-consuming and labour intensive and requires extensive training and technical skill of the experimenter, and it is invasive, precluding long-term recordings. This technique is also limited to a small number of cells per trial and it is usually necessary to change the patch pipette when going from one cell recording to the next one. The patch-clamp technique has been also extensively employed to study synaptic transmission by recording the electrical activity of the postsynaptic neurons in consequence of the stimulation of the presynaptic one. However, this approach does not allow to detect directly the release of neurotransmitter by the presynaptic cell and often requires the challenging task to record from two or more cells simultaneously. To this aim, multi-electrode arrays (MEAs) are one of the most viable devices to extracellularly record the electrical activity of a population of nearby cells. Moreover, MEAs can be used to electrically stimulate cells, thus allowing the interfacing between external electrical devices with a nervous tissue to be bidirectional. Similarly, artificial retinae are used for stimulation of neuronal tissue to regain a sense of vision. Finally, it is possible to design MEAs able to directly measure in real time the spatio-temporal dynamics of exocytosis. In the last two decades, much effort has been devoted to the realization of high-throughput bioprobes, targeted to provide, in real-time, non-invasive and multiparametric recordings of cell activity, using many different technologies and materials. This chapter will highlight the main outcomes of diamond-based probes, with a focus on micrographitized diamond MEAs. In more detail, we will describe their fabrication strategies and their suitability for real-time monitoring neurotransmitter release and action potential firing from cultured neurons.

### Why diamond?

In the field of neuroscience, MEAs represent a consolidated tool for electrical and chemical recording from in vitro cultures and from neuronal networks in vivo, as demonstrated by several examples of commercial devices already described in the literature. MEAs also promise to have many more exciting features in the future as the growing production of new prototypal sensors shows. Besides conventional materials [58], such as metals [101], silicon [68], polymers [38] and glass [67], diamond-based substrates became an attractive alternative during the last decade since they offer unique advantages due to the outstanding properties of this material, in terms of thermal conductivity, wide optical transparency, mechanical robustness and chemical inertness [63]. Furthermore, for the applications presented here, other peculiar properties of the diamond must be highlighted, like the optimal biocompatibility which is essential for performing long-term cultures [72], the lower absorption of organic molecules minimizing electrodes fouling [34], the chemically and electrochemically stability [112] and, above all, the possibility to control the electrical properties by means of doping [27] or inducing graphitization [62, 82], essential for the realization of conductive microelectrodes. In the following section, a detailed description of the technological strategies adopted to define the conductive path in the diamond is reported.

### MEA fabrication processes

#### Diamond doping

A widely adopted strategy for the fabrication of multi-electrode arrays is based on growing doped diamond layers, in which, by varying the doping level, a diamond can be tuned from insulator (10^−6^ S m^−1^) to a metal-like conductive material (10^4^ S m^−1^). In diamond, dopant elements employed are nitrogen [116] or more often boron [32, 42, 52, 65, 66] in concentration up to 10^21^ cm^−3^. Both of them are working as acceptors. MEA fabrication using doped diamond is based on a relatively simple workflow using both conventional lithographic steps and specific ones, necessary for the diamond growth, such as nanodiamond seeding. The former step includes the selection and the cleaning of a suitable carrier substrate that usually can be made of silicon, quartz or glass. Standard cleaning recipes suggest the use of acetone or alcohol followed by oxidizing solutions that ensure the complete removal of organic contamination and oxygen to terminate the surface of the wafers, optimizing the seeding procedure. For seeding, nanodiamonds (ND) which represent the nucleation centres for the nanocrystalline diamond film formation are spread onto the carrier substrate. A conventional technique for seeding with the desired pattern is photolithography, in which NDs are usually spin coated over the whole substrate and hard metal masks are used to protect areas of diamond nanoparticles from being etched away by a reactive ion etching process [49].

**Fig. 4:**
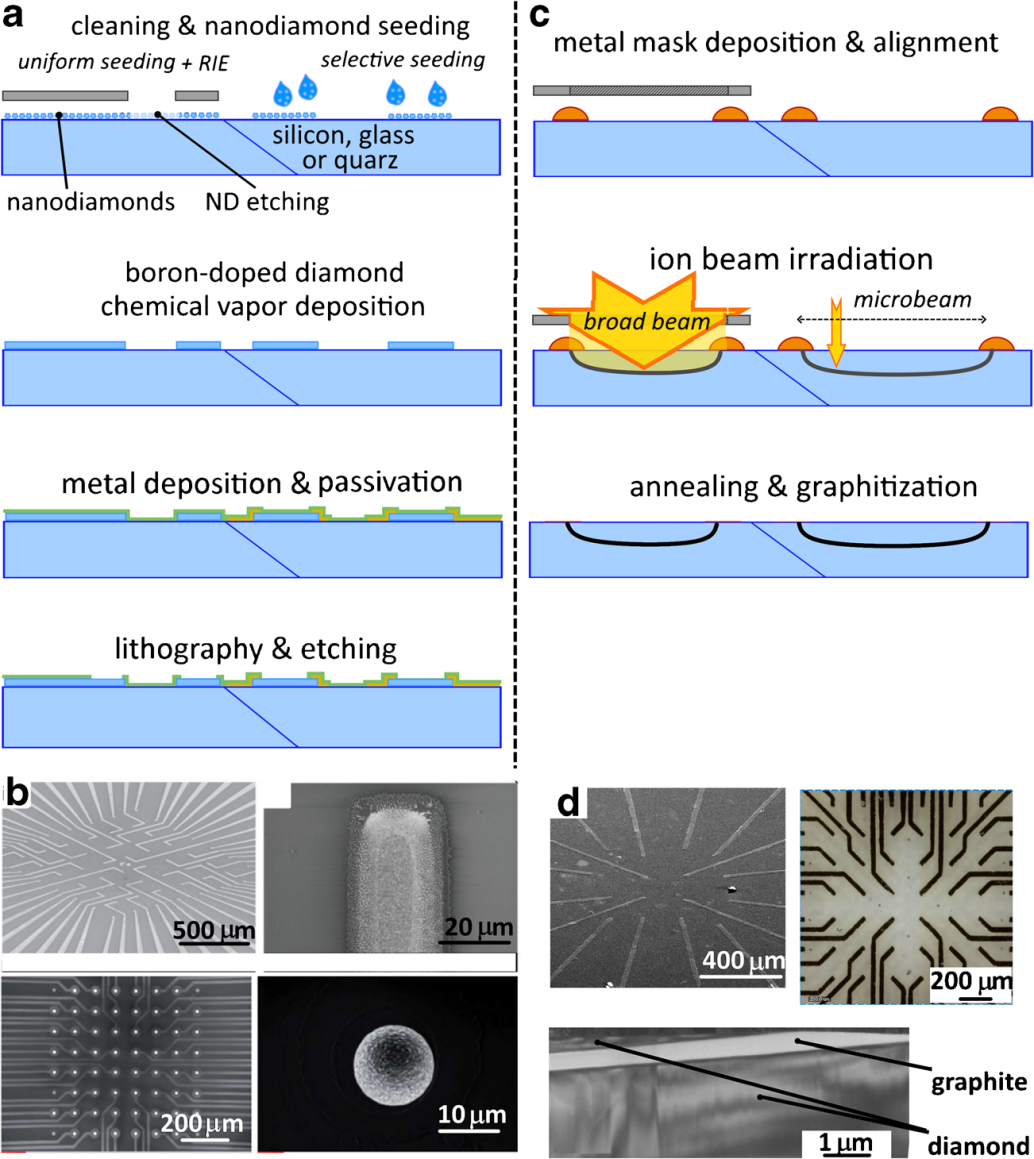
Processing steps for the fabrication of diamond-based MEAs. **a** Simplified workflow for boron-doped diamond growth: wafer substrate cleaning and nanodiamonds seeding steps, grown of a diamond by chemical vapour deposition, thin-film deposition of metal and insulating materials, lithography and etching steps. **b** Examples of boron-doped diamond MEAs: devices overview and magnification of the electrodes. Reproduced with permission from [42], Copyright Royal Society of Chemistry and [65], Copyright John Wiley and Sons. **c** Ion beam graphitization of diamond: diamond plates masking step, ion beam irradiation, thermal annealing for graphitization. **d** Examples of micrographitized diamond MEAs and TEM cross section of a graphitic electrode reaching the crystal surface embedded into the diamond matrix

An innovative solution is inkjet printing [98], where droplets of ND-based ink are directly deposited in specific places. The next step concerns the growth of the diamond layer by means of microwave plasma or hot-filament chemical vapour deposition: these techniques are based on the ionization of carbon precursors, provided by a flow of a gas mixture of H and > 1% CH_4_. The typical thicknesses used for the fabrication of electrodes ranged from a few tens up to a few hundreds of nanometres. In literature, two main strategies have been described for the electrical connection of the active regions of the electrode with the front-end electronics: the first one is based on the deposition of metal (Ti/Pt or Ti/Au) paths [49, 66] and the second one consists of forming directly the suitable paths with doped diamonds [65]. Finally, the electrodes must be passivated from the electrolyte solution using insulating materials such as standard resist like SU8 epoxy (thickness up to a few micrometres) or chemical deposited silicon nitride (Si_3_N_4_) [49], silicon oxide (SiO_2_) [49, 52] or intrinsic diamond [65, 66]. The fabrication steps are reported in Fig. 4.

#### Graphitization by ion beam lithography

An alternative approach for the fabrication of MEAs in diamond is the employment of ion beam lithography. This technique gives access to the properties of graphite, which are complementary to those of diamond (i.e. optically transparent/opaque and electrical insulator/conductor for diamond/graphite, respectively) exploiting the metastable nature of this latter carbon allotrope. For these reasons, several applications have been explored in literature realizing functional structures, including waveguides [57] or photonic structure [24], microfluidic channels [80] and other devices, such as micro-electromechanical systems [70] or ionizing radiation detectors [28]. Ion beams with energies from kilo-electron volts up to mega-electron volts are employed to promote the formation of structural damages within the diamond lattice, by inducing the formation of vacancies that promote the progressive amorphization of the crystal structure (a random network of sp3- and sp2-carbon atoms). In particular, a complete amorphization occurs if a critical threshold, usually called graphitization threshold (3 × 10^22^ ÷ 9 × 10^22^ cm^−3^; [10, 44]), is surpassed and therefore, the conversion of the highly damaged regions into graphite is allowed after high-temperature thermal annealing (> 900 °C, > 1 h in high vacuum or inert environment). This localized phase transition is used for the fabrication of electrically conductive graphitic structures embedded in the insulating diamond matrix. The induced effects of the graphitization process can be modelled using Monte Carlo simulations (i.e. SRIM, “Stopping Range of Ion in Matter” [121]) determining the profile of the damage density. Usually, vacancies are considered the main induced defects, and their distribution along the depth is evaluated as the product between the implantation fluence and the linear damage density. Input parameters for these simulations are the displacement energy for the carbon atom in diamond (50 eV), the density of the target material (3.52 g cm^−3^) and species and energy of the implanted ion. This fabrication technique can be applied both with polycrystalline or single-crystal artificial diamonds. These plates are purchasable from several companies providing high-quality substrates (type IIa optical grade—nitrogen and silicon defect with a concentration of less than 10 ppm) with an expense of few hundred Euro (depending on the size).

To manufacture micrographitized diamond MEAs, two different implantation approaches can be employed to define the three-dimensional damaged patterns, namely the microbeam or the broad beam irradiation. The former technique uses ion beams focused on the scale of micrometres (from 1 up to 10 μm) that are scanned over the sample defining the implanted region with a direct writing approach. The modulation of the ion penetration depth is defined by a degrading stack of metal layers placed directly over the sample surface, essential in order to have the graphitic structure endpoints emerging at the surface. The main limitations ascribable to this technology are the need for a microbeam line on an accelerator facility and the creation of the structures in a serial way [81].

**Fig. 5:**
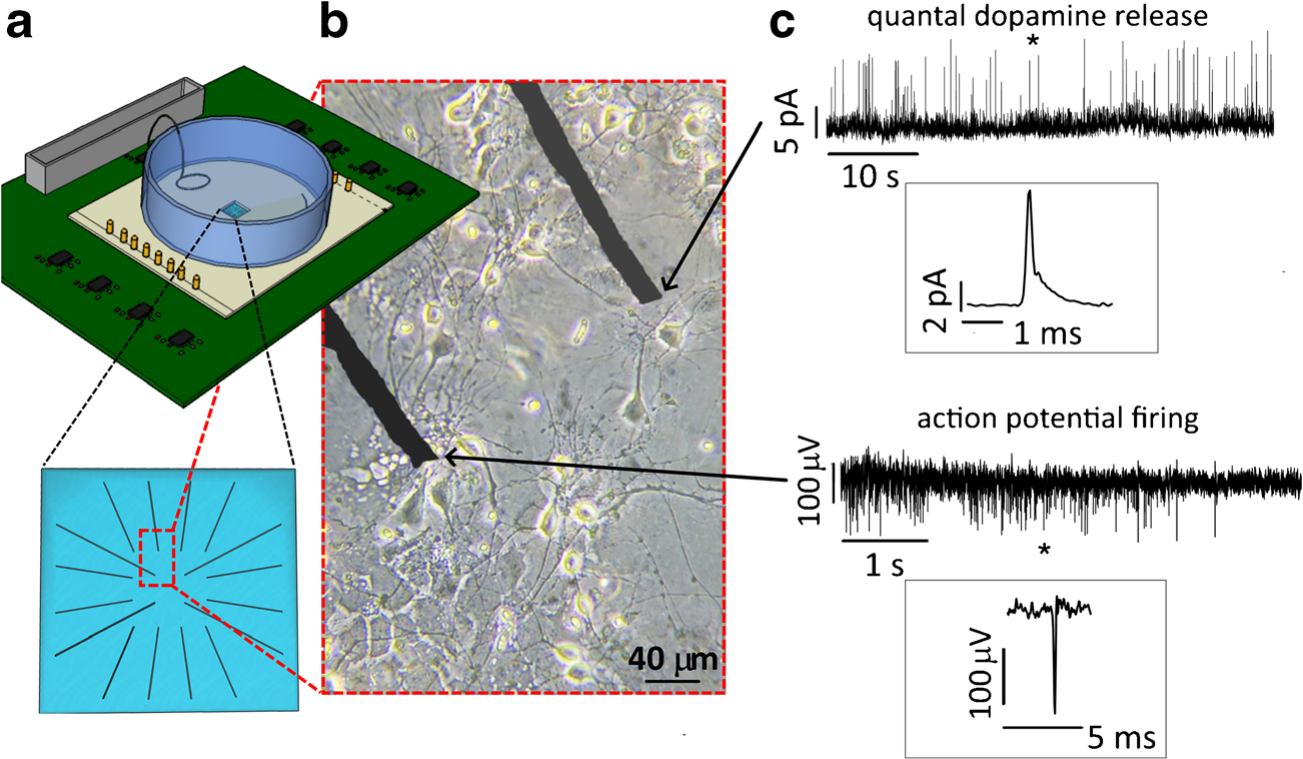
Micrographitized diamond MEA for simultaneous potentiometric and amperometric recording. **a** Scheme of the recording apparatus of micrographitized diamond MEA. Bottom: detail of the array geometry. **b** Midbrain neurons cultured on the micrographitized diamond MEA. **c** Simultaneous recordings of exocytosis and neuronal excitability using a micrographitized diamond MEA. Quantal dopamine release from midbrain dopaminergic neurons (upper trace) and action potential firing (lower trace) have been simultaneously detected from two graphitic electrodes of the 4 × 4 array. Insets show single events (amperometric spike and extracellular action potential, respectively) on a higher magnification scale

The latter approach takes advantage of the employment of an ion beam with a spot size larger than the sample surface. In this case, the lateral geometry of the electrodes is defined by the employment of a free-standing metal mask thick enough to completely stop the ion beam (few tens of micrometres for MeV ions). Also, in this case, the modulation of the penetration depth of the ions is controlled by metal deposition on the diamond surface. This approach allows implanting simultaneously multiple electrodes, fabricating a single sensor per irradiation, but a complex procedure is mandatory to properly align the two masks [77, 79, 107, 108]. Both approaches are schematically described in Fig. 5.

#### Electrical characterization and final assembly

Before the employment of the fabricated sensors for in vitro experiments, preliminary characterizations are usually performed to test their electrical and electrochemical properties. One of the most standard measurements is the current-voltage characterization, investigating both the electrical continuity and the resistance of the electrodes, confirming the absence of short circuits between the different electrodes or imperfections which might have occurred during the fabrication steps. Typical resistivities are ~ 10^14^ Ω cm for pristine diamond, ~ 10^−2^ Ω cm for doped diamond and ~ 10^−3^ Ω cm for graphitized diamond. Fabrication parameters, as the doping level, the electrode geometry and the interaction between the electrode materials with the physiological solution, influence the formation of the electric charge double layer and thereby affect the impedance of the electrode (resistive- and capacitive-dielectric properties) and therefore the performances of the sensors. Electrode impedance can be evaluated by impedance spectroscopy that employs electric testing protocols similar to the technique employed in the experiments, that is, the cyclic voltammetry. This electrochemical technique applies to the working electrode a sinusoidal voltage over a wide range of frequencies at a constant scan rate, in the forward and reverse direction for several times and measures the in-phase and out-of-phase current responses. The current flows within the potential window when the potential is swept, as the ions in the solution are moving to the electrode surface forming a double layer [87]. The charging and discharging of this double layer determine the background current within the potential window. This allows to gain information about several parameters, as the potential electrochemical window or the double-layer specific capacitance *C*_dl_, given by:

$$ {C}_{\mathrm{dl}}=\frac{1}{S}\frac{\Delta  i}{2v} $$

where *S* is the area of the electrode, *Δi* is the difference between the cathodic and anodic currents at zero bias and *v* is the potential scan rate.

After the characterization of the grown/modified diamond substrates and having verified the proper functionality, the final assembly of the crystal with the chip carrier can be performed. Typically, conductive (silver loaded) epoxy resin is employed, ensuring the electrical continuity between the microfabricated electrodes and the metal strips of the carrier, and successively, these connections are surrounded by insulating epoxy resin ensuring the passivation from the culture medium avoiding short circuits. The carrier is equipped with a perfusion chamber containing the growing medium essential for the cell plating. The device is finally interfaced with the front-end electronic whose performances and characteristics are determined by the kind of measurement performed (amperometry or potentiometry) but are in general similar to those of commercial systems like the amplifier for carbon fibre electrodes or titanium-nitride (TiN) MEAs [32, 79, 81, 109, 115].

### Mapping quantal exocytosis using doped and micrographitized diamond MEAs

Electrochemical detection of neurotransmitter release has been performed in the last decades by means of carbon fibre electrodes (CFE), considered the “gold standard” approach [115]. This methodology, useful for measuring real-time exocytosis from one cell at a time, requires CFEs to be positioned close to the cell surface and properly polarized, in order to oxidize the released molecules hitting the CFE surface. Oxidation of the molecules released during a unitary exocytotic event produces an amperometric current (spike), which is often preceded by a small current increase (foot). The high sensitivity of this approach, as well as its sub-millisecond temporal resolution, allows to distinguish the different phases of unitary exocytotic events, consisting of fusion pore opening, massive release during pore expansion and the consequent decline of vesicle content [3, 99]. Nevertheless, with the aim of improving the spatial resolution further beyond the CFE’s tip dimension, and achieving multi-site and subcellular detection of quantal exocytosis, microfabricated devices have been realized in noble metals [40], carbon nanotubes (CNTs), conductive polymers [119], transparent indium tin oxide [104] and carbon and diamond-based materials [49, 120]. Electrode arrays have been patterned either to be confined within the area of single cells [33, 50] or to be in contact with many cells [31, 49, 104]. Dating back to early 2000, the first nanofabricated electrochemical detector array was realized with four platinum electrodes. This array allowed to measure the spatio-temporal dynamics of exocytosis from a single chromaffin cell, by measuring the signals detected by the different electrodes [23].

Here, the application of similar MEAs will be shown, focusing on those realized in doped diamond or graphitic patterning. With the purpose of measuring subcellular exocytosis, high-density diamond-based MEAs have been fabricated using boron-doped diamond (BDD) grown on sapphire: initially, 4 and successively 9-rectangular electrodes were patterned within a circular opening of 20-μm diameter [19, 32]. We demonstrated that this patterning was suitable to detect micrometric hotspots, where exocytosis occurred, alternated by silent zones and allowed to map the spatial inhomogeneity of the secretory activity within a single chromaffin cell. In this configuration, the maximal resolution is determined by the area of the smallest electrode (12 μm^2^).

Besides detecting KCl-induced exocytosis from mouse and bovine chromaffin cells, the same BDD MEAs have been also employed to electrically stimulate exocytosis from chromaffin cells [32]. Successively, a different geometry has been realized, consisting of 12 round-shaped electrodes (2-μm diameter), grown on high-temperature glass and confined within a total area of ∼ 300 μm^2^ [19].

In a complementary application, which overlooks the subcellular spatial resolution, diamond-based MEAs have been patterned as low-density arrays, with the aim of simultaneously and independently recording electrochemical signals from cell populations. Such a configuration is primarily devoted to drug screening purposes and to reduce the time-consuming approach of single-cell measurements. Evidence of multi-site recordings from chromaffin and PC12 cells, as well as adrenal gland slices, come from doped [14] and micrographitized diamond MEAs, patterned as 4 × 4 arrays. In particular, micrographitized diamond MEAs have been demonstrated to successfully detect both the KCl-evoked exocytosis and the spontaneous catecholamine secretion. The rate of release and spike waveform was measured in the presence of variable external Ca^2+^ concentration (from 0.1 to 10 mM) [78]. As an example of an unprecedented achievement obtained by chip-based planar arrays, low-noise recordings performed by micrographitized diamond MEA allowed us to identify different waveforms of exocytotic spikes, including large, small and stand-alone foot events, whose amplitude does not exceed a few picoamperes (< 10 pA). These events, which may be interpreted as different modes of exocytosis (full fusion, kiss-and-run and kiss-and-stay, respectively), confirm, as a proof of principle, that the sensitivity of these devices is comparable with that of carbon fibre electrodes [111].

The micrographitized diamond MEA (16 channels), when used as amperometric sensors, have been employed to detect quantal release events not only from neuroendocrine chromaffin and PC12 cells [83], whose oxidation of granule content produces current events of hundreds of picoamperes and lasting tens of milliseconds, but also from cultured midbrain dopaminergic neurons: in this case, spike duration is completed within hundreds of microseconds [109]. Monitoring quantal exocytosis from dopaminergic neurons requires to increase the sampling frequency from 4 to > 25 kHz [109]. Spike half-time values have been estimated around 650 μs, in good agreement with those reported from CFEs [103]. Taking advantage of the excellent diamond biocompatibility, dopaminergic midbrain neurons have been cultured for approximately 3 weeks, and exocytosis was detected since 10 days after plating. Spontaneous release occurred at a very low frequency at physiological external Ca^2+^ concentration, while drastically increased using external 30 mM KCl as secretagogue. To the best of our knowledge, this is the first evidence showing the feasibility of multi-site detection of quantal exocytosis from neural cells. The possibility of performing multi-site detection of exocytosis from neurons opens new perspectives for screening the secretory properties of neural networks, which is specifically important to study if the synaptic transmission is impaired due to neurodegenerative diseases or other pathological conditions.

### Doped and micrographitized diamond MEAs to measure neuronal activity

Besides functioning as amperometric bioprobes, diamond-based MEAs have been interfaced with neurons, either for passively and non-invasively detecting their spontaneous electrical activity or for electrically stimulating the neuronal network [39]. Since these kinds of measures require long-term culturing, this goal can be achieved by taking advantage of diamond biocompatibility, which allows cell culturing for several days. Cultured rat hippocampal neurons, GT1-7 cells and chick ciliary ganglia neurons preserve their excitability when cultured on hydrogen- and oxygen-terminated diamond surfaces [9, 84], providing that the diamond probes are coated with adhesive molecules (poly-d-lysine, poly-dl-ornithine, laminin) to favour cell anchoring. Feasibility of recordings from in vitro and in vivo preparations have been shown using BDD-MEA and 3D-nanostructured BDD-MEA [49, 84], ultra-nanocrystalline nitrogen-doped diamond (NDD) MEA [116] and micrographitized diamond MEAs [109]. In the latter case, besides monitoring the spontaneous activity of midbrain neurons, we could provide a detailed analysis of the regulation of their firing properties by L-DOPA and the involvement of D_2_ autoreceptors. The potentiometric performance of micrographitized diamond MEAs (60 channels) is similar to the one of the traditional, commercially available MEAs (64 channels): the extracellularly recorded action potentials (APs) displayed comparable values, both for the mean amplitude (∼ − 50 μV) and the mean firing frequency (0.7 Hz ÷ 6.8 Hz). The same micrographitized diamond MEA has been proven to be suitable for measuring the spontaneous activity of the sino-atrial node and its modulation by BayK8644, confirming that data reproducibility is again comparable with the one of commercially available TiN MEAs [106]. Action potentials from contractile HL-1 cardiomyocyte-like cells were detected by BDD MEAs [65] and diamond transistor arrays [21], which preserved their properties even under mechanical stress-related conditions. All this evidence suggests that cell function is not hampered even after long-term culturing on diamond-based MEAs.

### Combining amperometric and potentiometric detection of neural signals

Different strategies have been pursued to carry out amperometric and potentiometric recordings using the same array. The versatility of diamond probes for performing amperometric and potentiometric recordings was tested by different groups. The feasibility of recording neuronal signals and electrochemical sensing has been exploited using an all-diamond probe, realized using undoped polycrystalline (poly-C) diamond film (as substrate) and a doped poly-C film for the sensing electrodes. In that case, electrochemical detection of applied noradrenaline was performed by means of cyclic voltammograms, taking advantage of the large electrochemical window of poly-C electrodes (from − 0.8 to + 1.4 V). The impedance of these electrodes is ~ 1.5 MΩ at 1 kHz (typical frequency of neural signals), and therefore, they can be successfully employed for recording neuronal activity [16].

More recently, carbon nanotube multi-electrode arrays (CNT-MEA) [105] have been demonstrated suitable to perform real-time measurements of dopamine release and electrophysiological responses, such as field postsynaptic potentials and action potentials from mouse hippocampal slices and rat cultured hippocampal neurons. Since neurotransmitter release was collected from striatal slices, the corresponding amperometric transients lasted several seconds. Nevertheless, according to our observations, the simultaneous detection of quantal dopamine release and action potential firing can be obtained by means of micrographitized diamond MEA [15]. The operating modality (amperometric versus potentiometric) was independently selected for each of the 16 electrodes. As shown in Fig. 5, following stimulation with external KCl, electrical spiking activity and quantal exocytotic release from dopaminergic neurons could be simultaneously detected by two electrodes of the array. In good agreement with values reported in the literature [103] and previous trials using micrographitized diamond MEA uniquely as amperometric probes, detected amperometric spikes had 0.60 ± 0.05 ms half-time width and 35.5 ± 1.9 pA unitary amplitude (*n* = 248 spikes). Simultaneous detection of action potential from another electrode of the array revealed events with 35 ± 1 μV peak amplitude (*n* = 152 events). These preliminary results confirm the potentiality of micrographitized diamond MEAs as sensitive bioprobes for performing multiparametric detection of neuronal activity.

## Voltage imaging of neuronal activity in brain slices and in vivo

The probes discussed so far are characterized by a very high temporal resolution, but their major drawback is still their limited spatial resolution. Although advanced microfabrication has led to a dramatic increase in the number and density of recording sites on the probes, an intrinsic limitation is represented by the unfavourable scaling of electrode impedance and signal-to-noise ratio with reduced electrode sizes and cross-talk among adjacent electrodes. However, to study complex brain functions, it is necessary to record simultaneously the electrical activity of as many neurons and their processes as possible. Voltage imaging can overcome the limitation of spatial resolution [35, 56, 75]. Importantly, voltage imaging can also be combined with patch techniques [2, 4, 5, 13, 30, 54, 73] and multi-electrode techniques [110].

The basic idea of voltage imaging is to convert the electrical activity of biological tissue into an optical signal and to image this optical signal. To detect fast voltage changes like action potentials, there are three types of probes available, able to convert the voltage changes over the membrane into an optical signal: The classic, synthetic, fast voltage-sensitive dyes, the genetically encoded voltage indicators (GEVIs) [51] and hybrid voltage sensors as a combination of genetic constructs and synthetic dyes [1, 45]. Imaging can be done with cameras attached to microscopes or with photomultiplier tubes integrated into scanning microscopes, like confocal or two-photon microscopes.

This section focuses on voltage imaging with fast voltage-sensitive dyes and two-photon microscopy.

### Fast voltage-sensitive dyes

**Fig. 6:**
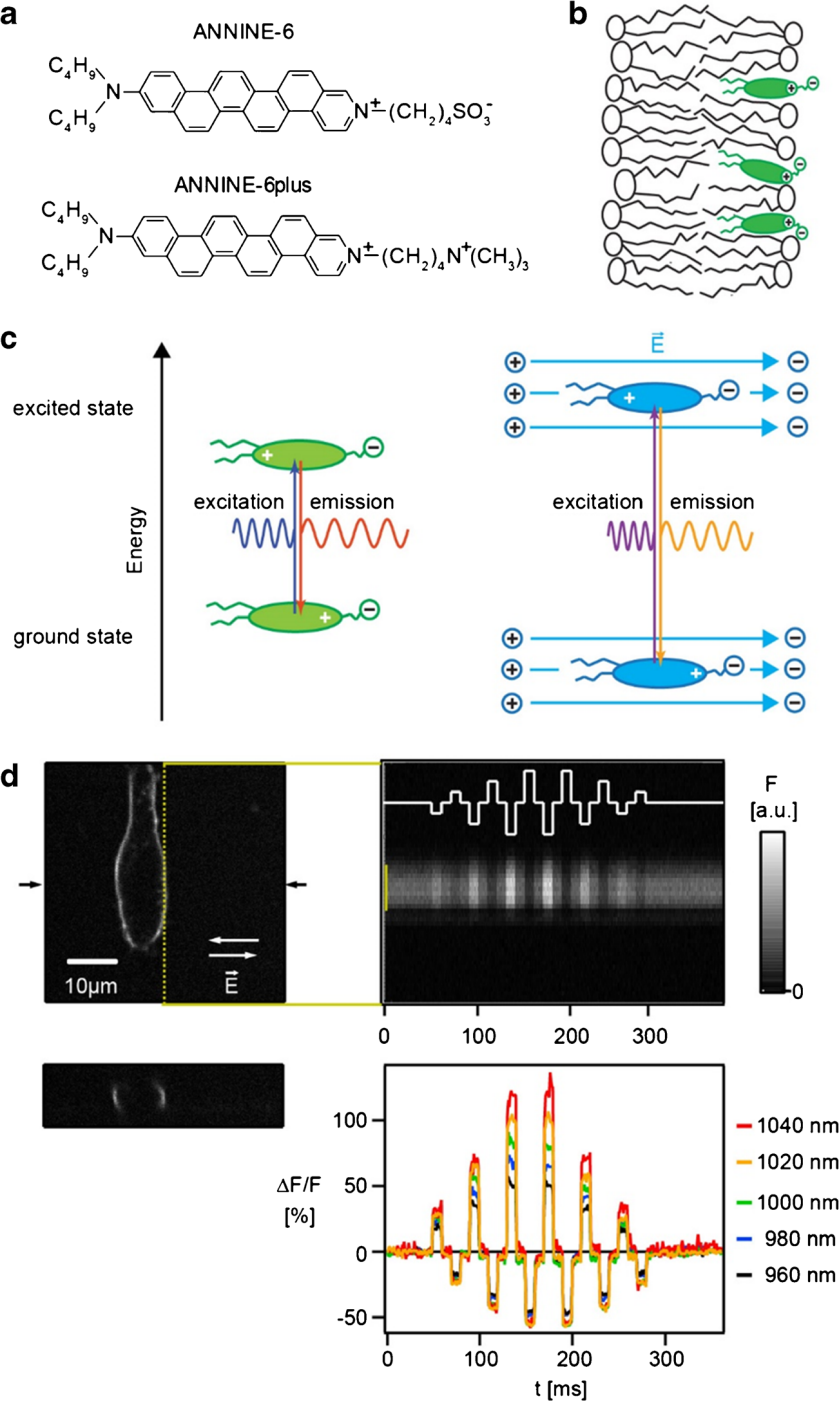
Mechanism of voltage sensitivity and voltage imaging. **a** Fast voltage-sensitive pure electrochromic dyes ANNINE-6 and ANNINE-6plus. ANNINE-6plus with two positive charges is more water soluble than ANNINE-6 with one positive and one negative charge. **b** Fast voltage-sensitive dyes are designed to bind to membranes in an ordered way. **c** A positive charge moves within the chromophore during the excitation and emission process (left). In the case of an external electric field, the charge must be moved in this external electric field, here against the electric field for the absorption process, and with the electric field during the emission process (right). **d** To convert the spectral shift into a detectable intensity change, the voltage-sensitive dye is excited at the spectral flank. Here, a HEK293 cell is labelled with ANNINE-6 (xy image, top left; xz-image, middle left) and a two-photon line scan is taken along the membrane (top right) at the location indicated (yellow dotted line). Additionally, an external electric field is applied (waveform, solid line). The intensity of the line scan changes accordingly. With increasing excitation wavelength, ANNINE-6 shows a larger relative fluorescence change for the same external electric field, with the highest sensitivity at the red spectral edge of absorption. Modified from [55, 56]

Fast voltage-sensitive dyes are known since the 1970s [17, 18] and were optimized ever since for different applications [2, 5, 30, 36, 46, 59, 60]. Fast voltage-sensitive dyes share a few design characteristics, here illustrated by two members of the ANNINE family (Fig. 6a). They have a hydrophobic carbohydrate tail, a hydrophobic chromophore and a hydrophilic headgroup. Due to this design, they bind to lipid membranes in an oriented way (Fig. 6b): The hydrophobic tail arrests in the core of the membrane and the headgroup aligns with the headgroups of the lipid molecules. An additional feature is the asymmetry of the chromophore. Due to the different configurations of the two nitrogen atoms in the chromophore, a positive net charge is located at one end of the chromophore. Interestingly, if the chromophore absorbs a photon, this net charge shifts within the chromophore. If the excited chromophore emits a photon, the charge moves back to its original position. Therefore, these dyes are also called charge-shift probes. Now, if the voltage-sensitive dye molecule is bound to a membrane, the charge shift within the molecule will interact with the electric field over the membrane (Fig. 6c). Typical resting potential of a neuron is − 60 mV in relation to the extracellular ground potential. During an action potential, the intracellular potential increases to + 40 mV. Important to remember is that the drop of the potential almost only occurs over the cell membrane and that the seemingly small voltage change during an action potential of 100 mV corresponds, due to the 5-nm-thin lipid membrane, to a 2 × 10^5^ V/cm electric field change.

How does an electric field over the voltage-sensitive dye modulate its fluorescence? The interaction between charge movement and the external electric field is best explained in an example: if the net charge relocates against the external electric field during the excitation process, more energy will be required to move it. Therefore, the absorption spectrum shifts to higher energy, i.e. towards the blue. In this case, also the emission spectrum shifts towards higher energy because the charge moves back to its original position with an additional push by the external electric field. So, for an ideal voltage probe, the excitation and emission spectrum will be shifted by the external electric field by the same energy [54]. As the spectral shift caused by the electric field corresponds to a shift in colour, these dyes are also called electrochromic dyes. Unfortunately, the spectral shift for the dyes discussed here is very small, in the range of a few nanometres for a 100-mV change of membrane potential. To convert the spectral shift into an intensity change, electrochromic dyes are not excited at the peak of the absorption spectrum but at the flank. If the spectrum shifts and the excitation intensity and wavelength stay constant, the number of absorbed photons will change, and therefore, also the fluorescence intensity will change. Importantly, the relative fluorescence change per voltage change, i.e. the fluorescence change normalized to the number of photons, increases towards the spectral edge of absorption [55].

This holds for one-photon excitation, i.e. regular excitation of a chromophore by absorption of one photon, as well as for two-photon excitation (Fig. 6d), where two photons of typically half the energy (equal to twice the wavelength) are absorbed simultaneously to excite the dye molecule [22, 106]. The relative fluorescence change is a measure of information about the voltage change normalized to the number of detected photons. If the information content of the detected photons is maximized, their number can be minimized. This is important because phototoxicity and bleaching, the two main obstacles of voltage imaging, are proportional to the number of excited dye molecules. So, by excitation of the voltage-sensitive dye at the red spectral edge of absorption, the relative fluorescence change is maximized and the number of excited dye molecules, and therefore phototoxicity and bleaching, is minimized. For example, by excitation at the red spectral edge of absorption with ANNINE-6, sensitivities of up to 50% per 100 mV can be reached [55].

Another important feature of electrochromic dyes is a linear and almost instantaneous (nanosecond range) response characteristics for voltage changes in the physiological range. The most popular dye of this kind, di4-ANEPPS and its derivatives are from the laboratory of Leslie M. Loew [59]. His lab also invented dyes for intracellular applications [4, 5], spectrally shifted voltage-sensitive dyes [118], voltage-sensitive dyes for two-photon excitation [118] and many others. Alternatively, ANNINE dyes can be used which were developed in the laboratory of Peter Fromherz based on elaborate physical-chemistry studies [25, 29, 30, 46, 54]. The ANNINE dyes are pure electrochromic probes [30, 46] and can be used in vitro [55, 73] and in vivo with one-photon and two-photon excitation [53, 95]. For a more detailed explanation of the mechanism of voltage sensing and protocols, specifically for the ANNINE dyes, see [56, 97] and [96], respectively.

### Labelling cells or tissue with synthetic voltage-sensitive dye in vitro or in vivo

The easiest way to label tissue with synthetic voltage-sensitive dye is to dissolve the hydrophobic dye in a solvent, like dimethyl sulfoxide (DMSO), dilute the solution in saline, and to bath apply it in vitro or inject it into the tissue in vivo. This protocol labels all cellular surfaces of neurons and glia, and the detected signal corresponds to the average membrane voltage change in the imaging volume. Voltage imaging from bulk-loaded tissue delivers therefore complementary data to extracellular recordings which detect current sources and sinks [110]. The optical voltage signals are typically below 1% relative fluorescence change.

To record voltage changes from single neurons and their processes, it is necessary to fill them with the dye by whole-cell patch clamping with dye in the pipette [4, 5] or by electroporation [95]. The dye diffuses intracellularly into the processes and allows voltage imaging at high spatio-temporal resolution. The detected signal reflects the membrane voltage change of the labelled structures. Compared with patch-clamp experiments, however, no absolute voltage measurements are so far possible. Also, the calibration of a relative fluorescence change to a voltage change is only possible in some neurons [13], as for example in Purkinje neurons [13, 95], because the dye labels also intracellular membranes. Therefore, the dye bound to these membranes contributes to the overall brightness but not to the signal, and since the labelling can vary locally, the calibration will fail. Finally, when imaging fine processes at the high spatio-temporal resolution, i.e. in the micrometre and millisecond range, the inherent optical noise becomes an important factor of the measurement and must be considered. Besides these limitations, an important advantage is the possibility to map the voltage changes in cells and in fine structures which are inaccessible to other methods.

**Fig. 7:**
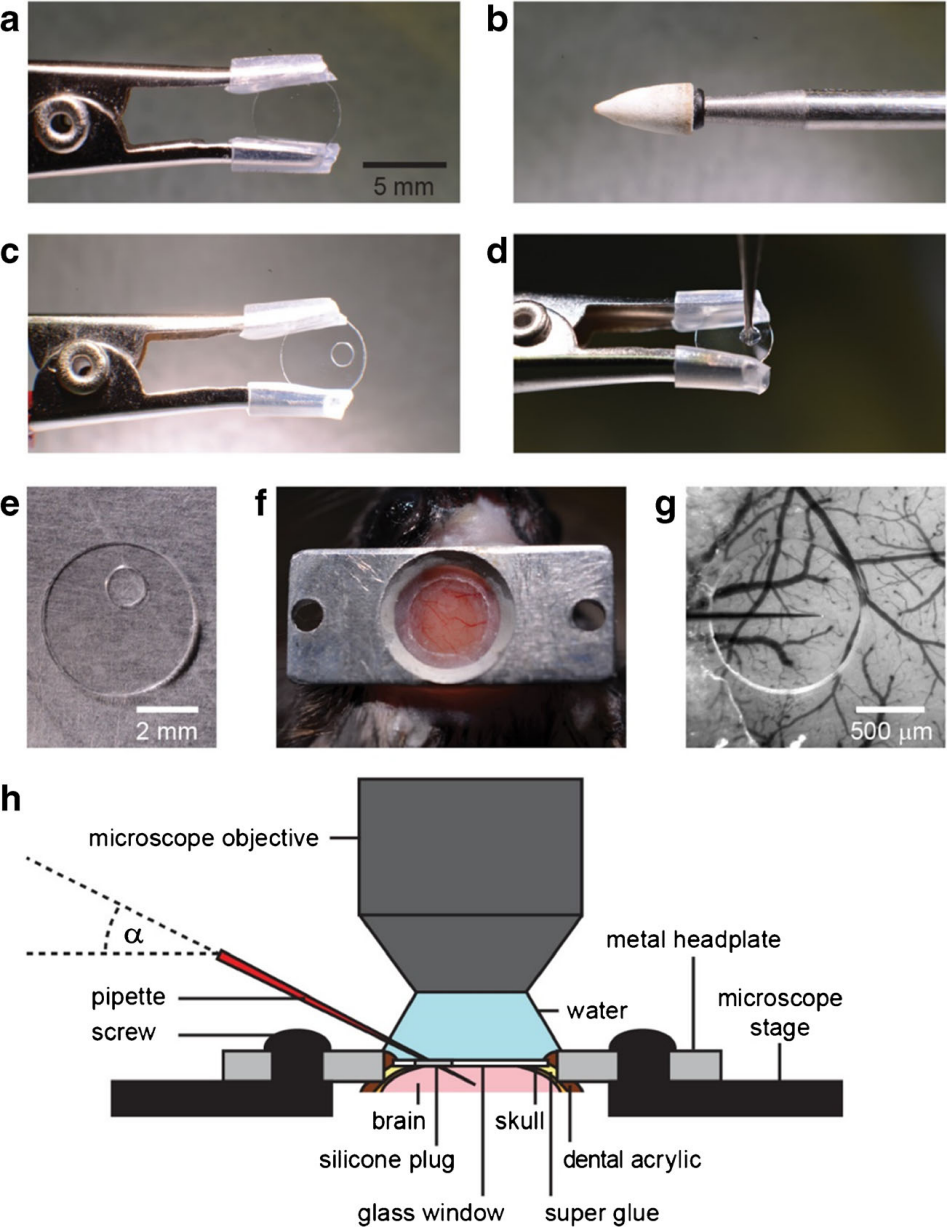
Chronic cranial window with an access port for dye injection, local drug application and/or electrophysiology. **a** A 5-mm round glass coverslip is held with a crocodile clamp. **b** With a conical grindstone drill bit, **c** a hole is drilled into the glass and **d** filled with PDMS. **e** After curing of the PDMS membrane, **f** the window with access port can be mounted on the craniotomy. **g** The blood vessel pattern can be seen through the glass and the PDMA membrane for positioning the pipette (coming from the left side) avoiding larger blood vessels. **h** Sketch of a typical arrangement for local injection of a dye, virus, drug or electrophysiology. Modified from [94]

For in vivo voltage imaging with single-cell resolution, it is crucial to have a chronic preparation with a cranial window [4]. There are several possibilities to make the brain accessible. The first one is to use a chronic cranial window with an access port (Fig. 7) [94, 96].

The thin silicone (polydimethylsiloxane, PDMS) membrane allows entering the brain repeatedly over several days with an injection pipette (Fig. 7g, h). The membrane is transparent and easy to penetrate, while the imaging area is under the glass and therefore stabilized for imaging even in awake, behaving animals. After retraction of the pipette, the silicone closes again, and no further procedure is required. The access port can also be used for local drug application and extracellular or cell-attached recordings. Interestingly, the glass window can be completely replaced by a PDMS membrane [43].

### Voltage imaging

In general, the aim of all fluorescence imaging methods is to excite fluorescent probes and to collect as many photons as possible which carry the information of interest. All the optical signals are afflicted by photon noise which is caused by the inherent fluctuation of photon flux. The photon flux follows Poisson statistics, and therefore, if *n* photons are detected on average, the standard deviation of the average will be $\sqrt{n}$. If we assume that the imaging setup is limited by photon noise (and most setups are), then the signal of interest S (relative fluorescence change) must overcome the photon noise to be clearly detected:

$$ S>\frac{\sqrt{n}}{n}=\frac{1}{\sqrt{n}} $$

Importantly, the relative photon noise $\frac{1}{\sqrt{n}}$ decreases with the number of detected photons. Based on this equation and the different labelling techniques, there are two imaging strategies applied: if the tissue is bulk loaded with a voltage-sensitive dye, the expected voltage signals are small because of the background fluorescence of cells not contributing to the voltage signal. In this case, many photons must be acquired to reach a noise level low enough to detect the signal. This is typically done by imaging with cameras but can also be achieved by two-photon microscopy if spatio-temporal averaging is used. Alternatively, if only specific cells are labelled with a voltage-sensitive dye, for example by electroporation, they can be imaged individually. In this case, most dye molecules are exposed to the membrane voltage change and therefore contribute to the optical voltage signal. As a result, the voltage signal S is larger and less photons are required for overcoming the relative photon noise. These relatively strong signals (compared with bulk-loaded tissue) allow imaging techniques employing cameras or image sectioning like confocal or two-photon microscopy. The latter has the advantage of overcoming, to some extent, the problem of light scattering and is therefore especially important for imaging in highly scattering tissue [22, 106].

### Wide-field voltage imaging

Voltage imaging with cameras of bulk-loaded tissue has a long and successful history [35, 37, 47]. Cameras were developed with the high quantum yield for high-speed photon detection. Also, these cameras, mainly charge-coupled devices (CCDs) and complementary metal-oxide-semiconductor (CMOS) cameras, were optimized for collecting hundreds of thousands or millions of electrons per pixel as a measure of detected photons, converting, roughly speaking, the photon noise, with a gain factor, into electron shot noise. In bulk-loaded tissue, high fluorescence intensities can be reached due to the abundance of dye molecules and the large imaging area. Therefore, low noise levels can be achieved. For example, if 10^6^ photons are detected per pixel per millisecond, the relative noise will be 1‰. This allows to image biological signals typically in the 0.1–1% range. As the biological samples are highly intermingled and all membrane surfaces are labelled, only average membrane voltage changes can be observed. Due to scattering and out of focus fluorescence, the resolution is in the range of tens or hundreds of micrometres.

### Two-photon voltage imaging

In two-photon microscopy [22, 106], pulsed infrared lasers are used to excite fluorescence only in the focus where the photon density is high enough to allow the simultaneous absorption of two photons, thereby adding up their energy to lift an electron into the excited state. The resulting fluorescence is indistinguishable from one-photon excitation. Two-photon microscopy has the advantage of being less sensitive to scattering than other imaging methods. To generate an image, the focal spot is directed through the sample by scan mirrors. This is typically done by galvo scanners or resonant scanners, with typical frame rates of 2 and 30 frames per second, respectively. This is too slow for imaging voltage changes in neurons, as an action potential lasts typically less than 1 ms in mammals.

#### Two-photon line scanning

The easiest solution to overcome this problem is to scan only the lines. Galvanometer scanners allow line scans in bidirectional mode (data acquisition during the forward and backward movement of the mirror) with frequencies of typically 2 kHz, while resonant scanning mirrors can reach line frequencies of typically 16 kHz. This is a simple and reliable method available on all two-photon microscopes to do one-dimensional voltage imaging. This is also applicable with confocal microscopy which can be used for thin or low-scattering samples. The evident disadvantage of line scans is one-dimensional measurement. Resonant scanning microscopes allow box scans with, for example 512 × 16 pixels in 1 ms, which is at least a very narrow image. Due to the high scan speed, the number of detected photons per pixel is low. By underfilling the back aperture of the objective lens, the spatial extent of excitation, especially in the axial direction (z-direction), can be increased and thereby the number of excited and detected photons.

**Fig. 8:**
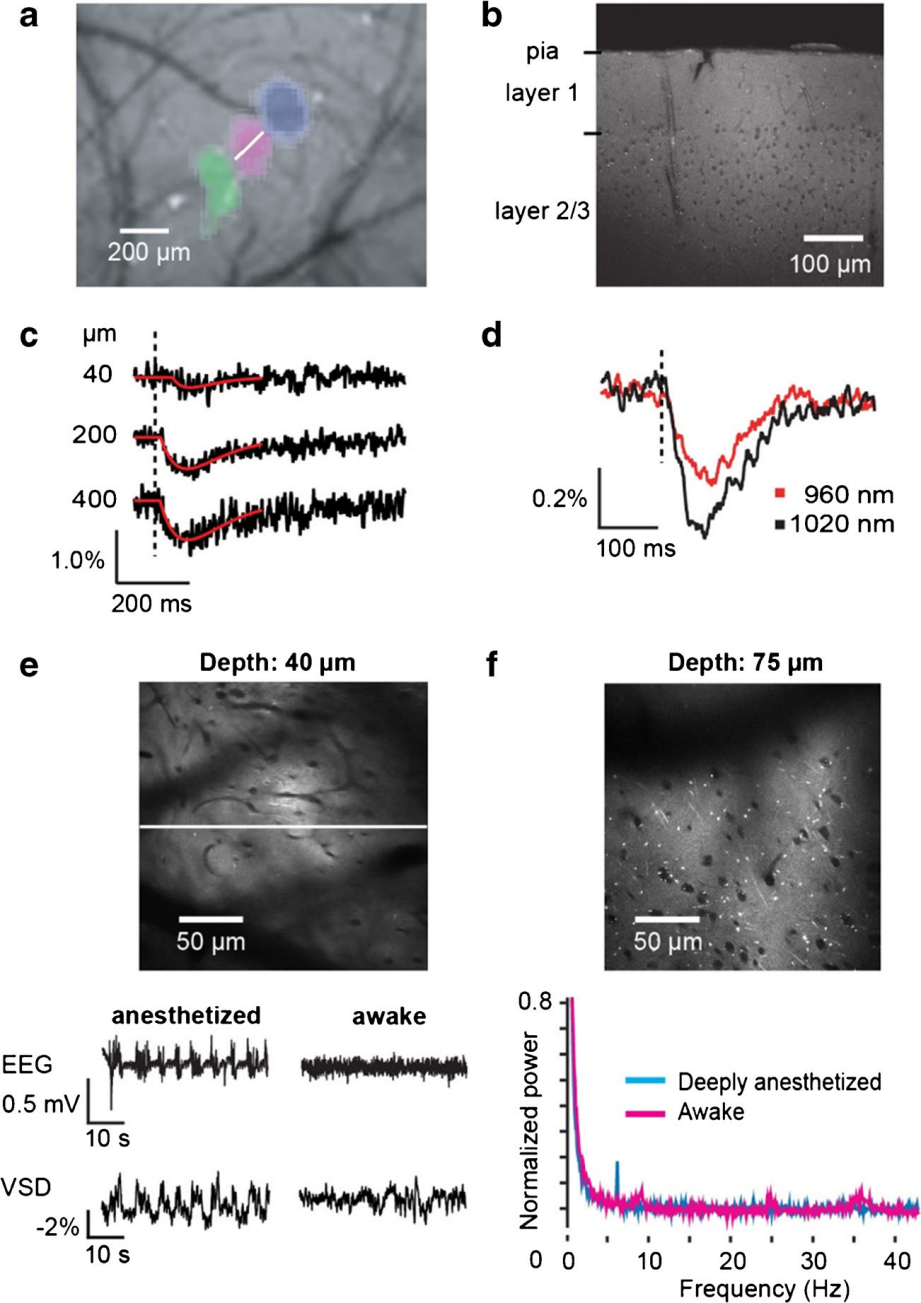
Two-photon voltage imaging of bulk-loaded tissue in vivo. **a** Imaging of intrinsic signals in the somatosensory cortex of the mouse after mechanical stimulation of vibrissae allows to determine the position of barrels. Different colours represent primary cortical areas responding to three adjacent vibrissae. **b** After injection of the voltage-sensitive dye ANNINE-6 at the location of the barrels, all surface membranes were labelled, with the somata remaining dark. The image was taken post-mortem in a brain slice. **c** Line scans at the position indicated in **a** (white line) can be performed depth resolved, showing the average membrane depolarization in response to vibrissa stimulation. If ANNINE-6 is applied to the extracellular leaflet of the plasma membrane, a depolarization results in a decrease of intensity. **d** By changing the excitation wavelength, the averaged signal increases towards the red spectral edge of absorption as shown in Fig. 6d. **e** To measure cortical oscillations, ANNINE-6 was injected into the tissue, and line scans were taken in the anaesthetized and awake mouse. Simultaneously, the electroencephalogram (EEG) was measured, to compare EEG and voltage imaging. Spindle activity can be seen in both measurements. The EEG shows biphasic signals during the spindles while the voltage imaging shows a depolarization (negative fluorescence change). **f** A new member of the ANNINE dye family is di1-ANNINE-6plus which diffuses well into layer 1 from the brain surface. It allows to measure frequency spectra spatially resolved. Here, as an example, layer 1 shows several oscillation bands, including a low gamma band in the awake state (36 Hz) which disappear under deep anaesthesia. Modified from [20, 53]

Two-photon line scanning in combination with bulk loading allows to image average membrane potentials depth resolved (Fig. 8). For example, to study barrel cortex of the mouse, the location of primary responses can be determined by imaging of intrinsic signals (Fig. 8a) [12, 53]. This information can be used for targeted voltage-sensitive dye injection, for example into cortex, resulting in locally homogeneous labelling (Fig. 8b). The range of labelling can be adjusted by the number of injections and the injection volume. Two-photon line scanning now allows measuring average membrane voltage changes depth resolved (Fig. 8c). As the average membrane voltage changes are small, spatio-temporal averaging is required, and/or the averaging of trials. To show that the recorded signal is a voltage signal, the excitation wavelength can be changed (Fig. 8d). As expected from a pure electrochromic dye [54] and as shown in cell cultures (Fig. 6d) [55], the signal amplitude changes due to the excitation wavelength-dependent sensitivity. In the same preparation, it is possible to analyse local membrane voltage oscillations by spatio-temporal averaging (Fig. 8e). Sleep spindles, here induced by anaesthesia, can be easily detected with two-photon voltage imaging. When the animal wakes up, cortical activity decorrelates and the oscillations disappear [53].

An alternative to bulk loading by injection into the tissue is to apply a dye which diffuses into the tissue [35], as the new derivative of ANNINE dyes, di1-ANNINE-6plus [20]. In combination with a new way to apply dye solution in large quantities (several microliters) locally and without removing the dura [20], di1-ANNINE-6plus can be used to study oscillations specifically in cortical layer 1 (Fig. 8f) [20].

**Fig. 9:**
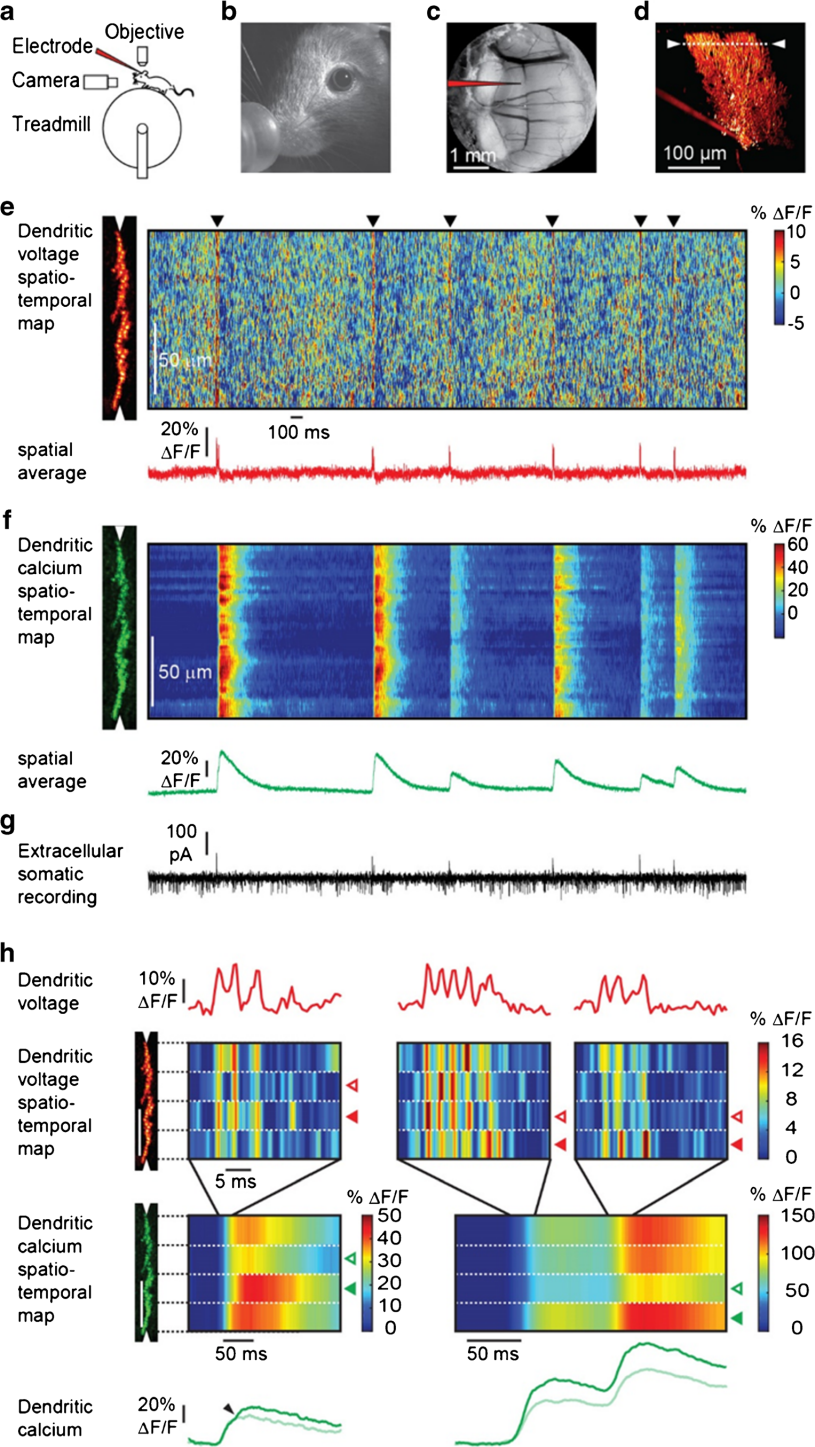
Simultaneous voltage and calcium imaging of Purkinje neuron dendrites and somatic recording in the awake mouse after intracellular application of ANNINE-6plus. **a** Sketch of the setup. **b** Experiment was done in an awake mouse with chronic cranial window **c** with access port after electroporating, **d** a single Purkinje neuron with ANNINE-6plus. **e** A line scan at 2 kHz was taken along the Purkinje neuron dendrites (scan position shown in **d**) to record a voltage spatio-temporal map in an awake mouse. The spatially averaged dendritic voltage (red trace) clearly shows dendritic complex spikes (black triangles). **f** The corresponding dendritic calcium spatio-temporal map and spatially averaged dendritic calcium (green trace) measured with the genetically encoded calcium indicator GCaMP6f shows large calcium transients for every dendritic complex spike. **g** The access port allowed simultaneous extracellular electrical recordings from the soma (black trace) while imaging voltage and calcium transients from the dendrites. Simple spikes (somatic Na^+^ spikes) result in a current sink at the soma, while dendritic complex spikes (dendritic Ca^2+^ spikes) result in a dominant current source signal at the soma. **h** Different parts of the dendritic tree show a different number of spikelets during the same complex spike event. The number of spikelets correlates with the amplitude of the calcium transients in each part of the dendritic tree. Open red arrowheads indicate spatially localized low activity, filled with red arrowheads and show high activity. Spatially localized dendritic spikelets during dendritic complex spikes correlate with a local boost in the dendritic calcium transient (green, filled arrowheads). Modified from [95]

If the voltage-sensitive dye is filled into a single neuron, for example by electroporation in vivo (Fig. 9a–c) [95], the target neurons can be reconstructed with two-photon microscopy (Fig. 9d) and used for recording in awake, behaving animals. In the case of the fan-like dendrite of Purkinje neurons, it is possible to scan along the dendrite and image simultaneously voltage (Fig. 9e) and calcium (Fig. 9f).

Additionally, electrical activity can be recorded extracellularly from the soma (Fig. 9g). Dendritic complex spikes can be easily detected in all three measurements (Fig. 9e–g). Interestingly, also subthreshold postsynaptic potentials can be detected as local depolarizations (red hotspots in Fig. 9e). The signal-to-noise ratio even reveals differences in the number of spikelets (Fig. 9h).

#### Bessel beam excitation, random-access scanning and fast two-photon imaging

If dendrites of single neurons are the target for voltage imaging, line scanning becomes difficult because of the 3-dimensional, curved morphology, changing direction every few micrometres. Instead of just elongating the point spread function of excitation by underfilling the back focal plane, it is possible to extend the point spread function by Bessel beam excitation for up to a few hundred micrometres [61]. Thereby, labelled 3-dimensional neurons are projected into a single 2-dimensional plane. However, if the labelling is too dense, signals will be mixed due to the projection. An alternative is random-access scanning. This allows scanning along with 3-dimensional structures with the help of acousto-optic deflectors. The result is again a line scan but in this case along an almost arbitrary 2-dimensional or 3-dimensional path [88].

Finally, maybe one of the most promising methods is to use an all-optical trick to scan up to 3000 frames per second full frame [117]. In this method, the excitation laser is split up into multiple sub-pulses and thereby generating an array of spatially separated and temporally delayed foci in the focal plane of the microscope objective. By pulse gated, fast sampling, frame rates in the kilohertz range can be reached.

## Outlook

This paper describes the specific advancements of patch-clamp recording, multi-electrode recording and voltage imaging during the last decades. Several challenges still remain:

Regarding the patch-clamp technique, it would be major progress to have automatic systems based on artificial intelligence able to reliably record not only from cells in culture or oocytes expressing membrane channels but from cells mechanically isolated from tissue or still inserted in it. Another major improvement would be to develop three-dimensional microprinting of patch pipette matrices made with new materials that reliably attain seals, so as to have the possibility to record simultaneously in whole-cell mode from many neurons of nerve tissue.

An important goal for multi-electrode arrays is to use them chronically in the human brain, to interface with external devices, for instance, to move myoelectric prosthesis or stimulate the brain with artificial sensors. The major, still unresolved issue when using these probes, is the progressive encapsulation of the probe by a glial insulating scar (a process called gliosis) and neuronal migration far from the probe [26], most likely an immune response triggered by probe implantation. This causes the progressive deterioration of the communication between the probe and the neuronal tissue. Also, problems occurring due to the relative shear motion between the stiff probe and the (soft) brain tissue, the chemical instability and delamination of electrodes have to be addressed [114]. Therefore, it is mandatory in the future to develop probes that have a stiffness comparable with the brain tissue and that are at the same time brain tissue compatible with not trigger any inflammatory processes [76].

The power of voltage imaging could be further improved by developing more sensitive and biocompatible dyes. Most importantly, the problem of labelling specific cell types in vitro and in vivo has to be addressed, for example by genetic targeting of synthetic dyes [45]. Finally, fast, high quantum yield cameras, faster scanning systems and light-sheet or holographic illumination techniques could strongly advance the field.

Based on the current dynamics of the field, there are exciting times ahead, with many new and improved techniques to come, which will help to reveal some of the secrets of the brain.

## References

[1] Abdelfattah AS, Kawashima T, Singh A, Novak O, Liu H, Shuai Y, Huang YC, Campagnola L, Seeman SC, Yu J, Zheng J, Grimm JB, Patel R, Friedrich J, Mensh BD, Paninski L, Macklin JJ, Murphy GJ, Podgorski K, Lin BJ, Chen TW, Turner GC, Liu Z, Koyama M, Svoboda K, Ahrens MB, Lavis LD, Schreiter ER (2019). Bright and photostable chemigenetic indicators for extended in vivo voltage imaging. Science.

[2] Acker CD, Loew LM (2013). Characterization of voltage-sensitive dyes in living cells using two-photon excitation. Methods Mol Biol.

[3] Álvarez de Toledo G, Montes MÁ, Montenegro P, Borges R (2018). Phases of the exocytotic fusion pore. FEBS Lett.

[4] Antic S, Major G, Zecevic D (1999). Fast optical recordings of membrane potential changes from dendrites of pyramidal neurons. J Neurophysiol.

[5] Antic S, Zecevic D (1995). Optical signals from neurons with internally applied voltage-sensitive dyes. J Neurosci.

[6] Aquila M, Benedusi M, Fasoli A, Rispoli G (2014). Pressure-polished borosilicate pipettes are “universal sealer” yielding low access resistance and efficient intracellular perfusion. Methods Mol Biol.

[7] Aquila M, Benedusi M, Fasoli A, Rispoli G (2015). Characterization of zebrafish green cone photoresponse recorded with pressure-polished patch pipettes, yielding efficient intracellular dialysis. PLoS One.

[8] Aquila M, Dell’Orco D, Fries R, Koch KW, Rispoli G (2019). Incorporating phototransduction proteins in zebrafish green cone with pressure-polished patch pipettes. Biophys Chem.

[9] Ariano P, Lo Giudice A, Marcantoni A, Vittone E, Carbone E, Lovisolo D (2009). A diamond-based biosensor for the recording of neuronal activity. Biosens Bioelectron.

[10] Battiato A, Lorusso M, Bernardi E, Picollo F, Bosia F, Ugues D, Zelferino A, Damin A, Baima J, Pugno NM, Ambrosio EP, Olivero P (2016). Softening the ultra-stiff: controlled variation of Young’s modulus in single-crystal diamond by ion implantation. Acta Mater.

[11] Benedusi M, Aquila M, Milani A, Rispoli G (2011). A pressure-polishing set-up to fabricate patch pipettes that seal on virtually any membrane, yielding low access resistance and efficient intracellular perfusion. Eur Biophys J.

[12] Bonhoeffer T, Grinvald A, Toga AW, Mazziotta JC (1996). Optical imaging based on intrinsic signals: the methodology. Brain mapping: the methods.

[13] Canepari M, Saggau P, Zecevic D, Canepari M, Zecevic D (2010). Combined voltage and calcium imaging and signal calibration. Membrane potential imaging in the nervous system.

[14] Carabelli V, Gosso S, Marcantoni A, Xu Y, Colombo E, Gao Z, Vittone E, Kohn E, Pasquarelli A, Carbone E (2010). Nanocrystalline diamond microelectrode arrays fabricated on sapphire technology for high-time resolution of quantal catecholamine secretion from chromaffin cells. Biosens Bioelectron.

[15] Carabelli V, Tomagra G, Bonardi M, Picollo F, Picconi B, Pasquarelli A, Olivero P, Calabresi P, Carbone E, Marcantoni A (2019) 16^th^ ANA-25^th^ APHAR Meeting, 80:#152.

[16] Chan H-Y, Aslam DM, Wang SH, Swain GM, Wise KD (2008) Fabrication and testing of a novel all-diamond neural probe for chemical detection and electrical sensing applications. In: 2008 IEEE 21st International Conference on Micro Electro Mechanical Systems. IEEE, pp 244–247

[17] Cohen LB, Salzberg BM (1978). Optical measurement of membrane potential. Rev Physiol Bioch P.

[18] Cohen LB, Salzberg BM, Davila HV, Ross WN, Landowne D, Waggoner AS, Wang CH (1974). Changes in axon fluorescence during activity - molecular probes of membrane potential. J Membr Biol.

[19] Colombo E, Men Y, Scharpf J, Pietzka C, Dipalo M, Herfurth P, Gao Z, Schneider M, Carabelli V, Carbone E, Kohn E, Pasquarelli A (2011). Fabrication of a NCD microelectrode array for amperometric detection with micrometer spatial resolution. Diam Relat Mater.

[20] Dalphin N, Dorgans K, Khaskin E, Kuhn B (2020). Voltage imaging of cortical oscillations in layer 1 with two-photon microscopy. eNeuro.

[21] Dankerl M, Eick S, Hofmann B, Ingebrandt MHS, Offenhäusser A, Stutzmann M, Garrido JA (2009). Diamond transistor array for extracellular recording from electrogenic cells. Adv Funct Mater.

[22] Denk W, Strickler JH, Webb WW (1990). Two-photon laser scanning fluorescence microscopy. Science.

[23] Dias AF, Dernick G, Valero V, Yong MG, James CD, Craighead HG, Lindau M (2002). An electrochemical detector array to study cell biology on the nanoscale. Nanotechnology.

[24] Drumm VS, Alves ADC, Fairchild BA, Ganesan K, McCallum JC, Jamieson DN, Prawer S, Rubanov S, Kalish R, Feldman LC (2011). Surface damage on diamond membranes fabricated by ion implantation and lift-off. Appl Phys Lett.

[25] Ephardt H, Fromherz P (1993). Fluorescence of amphiphilic hemicyanine dyes without free double-bonds. J Phys Chem.

[26] Fernández E, Greger B, Aranda I, Botella C, Albisua J, Soto-Sánchez C, Alfaro A, Normann RC, House PA (2014). Acute human brain responses to intracortical microelectrode arrays: challenges and future prospects. Front Neuroeng.

[27] Fontaine F, Gheeraert E, Deneuville A (1996). Conduction mechanisms in boron implanted diamond films. Diam Relat Mater.

[28] Forneris J, Grilj V, Jakšić M, Lo Giudice A, Olivero P, Picollo F, Skukan N, Verona C, Verona-Rinati G, Vittone E (2013). IBIC characterization of an ion-beam-micromachined multi-electrode diamond detector. Nucl Instruments Methods Phys Res Sect B Beam Interact with Mater Atoms.

[29] Fromherz P (1995). Monopole-dipole model for symmetrical solvatochromism of hemicyanine dyes. J Phys Chem.

[30] Fromherz P, Hübener G, Kuhn B, Hinner MJ (2008). ANNINE-6plus, a voltage-sensitive dye with good solubility, strong membrane binding and high sensitivity. Eur Biophys J Biophy.

[31] Gillis KD, Liu XA, Marcantoni A, Carabelli V (2018). Electrochemical measurement of quantal exocytosis using microchips. Pflugers Arch4.

[32] Gosso S, Turturici M, Franchino C, Colombo E, Pasquarelli A, Carbone E, Carabelli V (2014). Heterogeneous distribution of exocytotic microdomains in adrenal chromaffin cells resolved by high-density diamond ultra-microelectrode arrays. J Physiol.

[33] Granado TC, Neusser G, Kranz C, Filho JBD, Carabelli V, Carbone E, Pasquarelli A (2015). Progress in transparent diamond microelectrode arrays. Phys Status Solidi.

[34] Grieten L, Janssens SD, Ethirajan A, Vanden BN, Ameloot M, Michiels L, Haenen K, Wagner P (2011). Real-time study of protein adsorption on thin nanocrystalline diamond. Phys Status Solidi Appl Mater Sci.

[35] Grinvald A, Hildesheim R (2004). VSDI: a new era in functional imaging of cortical dynamics. Nat Rev Neurosci.

[36] Grinvald A, Hildesheim R, Farber IC, Anglister L (1982). Improved fluorescent probes for the measurement of rapid changes in membrane potential. Biophys J.

[37] Grinvald A, Lieke E, Frostig RD, Gilbert CD, Wiesel TN (1986). Functional architecture of cortex revealed by optical imaging of intrinsic signals. Nature.

[38] Guo L, Ma M, Zhang N, Langer R, Anderson DG (2014). Stretchable polymeric multielectrode array for conformal neural interfacing. Adv Mater.

[39] Hadjinicolaou AE, Leung RT, Garrett DJ, Ganesan K, Fox K, Nayagam DAX, Shivdasani MN, Meffin H, Ibbotson MR, Prawer S, O’Brien BJ (2012). Electrical stimulation of retinal ganglion cells with diamond and the development of an all diamond retinal prosthesis. Biomaterials.

[40] Hafez I, Kisler K, Berberian K, Dernick G, Valero V, Yong MG, Craighead HG, Lindau M (2005). Electrochemical imaging of fusion pore openings by electrochemical detector arrays. Proc Natl Acad Sci U S A 2005.

[41] Hamill OP, Marty A, Neher E, Sakmann B, Sigworth FJ (1981). Improved patch-clamp techniques for high-resolution current recording from cells and cell-free membrane patches. Pflugers Arch.

[42] Hébert C, Scorsone E, Bendali A, Kiran R, Cottance M, Girard HA, Degardin J, Dubus E, Lissorgues G, Rousseau L, Mailley P, Picaud S, Bergonzo P (2014). Boron doped diamond biotechnology: from sensors to neurointerfaces. Faraday Discuss.

[43] Heo C, Park H, Kim YT, Baeg E, Kim YH, Kim SG, Suh M (2016). A soft, transparent, freely accessible cranial window for chronic imaging and electrophysiology. Sci Rep.

[44] Hickey DP, Jones KS, Elliman RG (2009). Amorphization and graphitization of single-crystal diamond - a transmission electron microscopy study. Diam Relat Mater.

[45] Hinner MJ, Hübener G, Fromherz P (2004). Enzyme-induced staining of biomembranes with voltage-sensitive fluorescent dyes. J Phys Chem B.

[46] Hübener G, Lambacher A, Fromherz P (2003). Anellated hemicyanine dyes with large symmetrical solvatochromism of absorption and fluorescence. J Phys Chem B.

[47] Iijima T, Witter MP, Ichikawa M, Tominaga T, Kajiwara R, Matsumoto G (1996). Entorhinal-hippocampal interactions revealed by real-time imaging. Science.

[48] Johnson BE, Brown AL, Goodman MB (2008). Pressure-polishing pipettes for improved patch-clamp recording. J Vis Exp.

[49] Kiran R, Rousseau L, Lissorgues G, Scorsone E, Bongrain A, Yvert B, Picaud S, Mailley P, Bergonzo P (2012). Multichannel boron doped nanocrystalline diamond ultramicroelectrode arrays: design, fabrication and characterization. Sensors (Switzerland).

[50] Kisler K, Kim BN, Liu X, Berberian K, Fang Q, Mathai CJ, Gangopadhyay S, Gillis KD, Lindau M (2012). Transparent electrode materials for simultaneous amperometric detection of exocytosis and fluorescence microscopy. J Biomater Nanobiotechnol.

[51] Knöpfel T (2012). Genetically encoded optical indicators for the analysis of neuronal circuits. Nat Rev Neurosci.

[52] Krůšek J, Dittert I, Smejkalová T, Kořínek M, Gottfriedová K, Freislebenová H, Neuhöferová E, Klimša L, Sedláková S, Taylor A, Mortet V, Petrák V, Benson V, Petráková V (2019). Molecular functionalization of planar nanocrystalline and porous nanostructured diamond to form an interface with newborn and adult neurons. Phys Status Solidi.

[53] Kuhn B, Denk W, Bruno RM (2008). In vivo two-photon voltage-sensitive dye imaging reveals top-down control of cortical layers 1 and 2 during wakefulness. Proc Natl Acad Sci U S A.

[54] Kuhn B, Fromherz P (2003). Anellated hemicyanine dyes in a neuron membrane: molecular Stark effect and optical voltage recording. J Phys Chem B.

[55] Kuhn B, Fromherz P, Denk W (2004). High sensitivity of Stark-shift voltage-sensing dyes by one- or two-photon excitation near the red spectral edge. Biophys J.

[56] Kuhn B, Roome CJ (2019). Primer to voltage imaging with ANNINE dyes and two-photon microscopy. Front Cell Neurosci.

[57] Lagomarsino S, Olivero P, Bosia F, Vannoni M, Calusi S, Giuntini L, Massi M (2010). Evidence of light guiding in ion-implanted diamond. Phys Rev Lett.

[58] Lemaître F, Guille Collignon M, Amatore C (2014). Recent advances in electrochemical detection of exocytosis. Electrochim Acta.

[59] Loew LM, Cohen LB, Dix J, Fluhler EN, Montana V, Salama G, Wu JY (1992). A naphthyl analog of the aminostyryl pyridinium class of potentiometric membrane dyes shows consistent sensitivity in a variety of tissue, cell, and model membrane preparations. J Membr Biol.

[60] Loew LM, Simpson LL (1981). Charge-shift probes of membrane potential - a probable electrochromic mechanism for para-aminostyrylpyridinium probes on a hemispherical lipid bilayer. Biophys J.

[61] Lu R, Sun W, Liang Y, Kerlin A, Bierfeld J, Seelig JD, Wilson DE, Scholl B, Mohar B, Tanimoto M, Koyama M, Fitzpatrick D, Orger MB, Ji N (2017). Video-rate volumetric functional imaging of the brain at synaptic resolution. Nat Neurosci.

[62] Lühmann T, Wunderlich R, Schmidt-Grund R, Barzola-Quiquia J, Esquinazi P, Grundmann M, Meijer J (2017). Investigation of the graphitization process of ion-beam irradiated diamond using ellipsometry, Raman spectroscopy and electrical transport measurements. Carbon N Y.

[63] Nemanich RJ, Carlisle J a, Hirata A, Haenen K (2014). CVD diamond—research, applications, and challenges. MRS Bull.

[64] Malboubi M, Gu Y, Jiang K (2011). Surface properties of glass micropipettes and their effect on biological studies. Nanoscale Res Lett 2011.

[65] Maybeck V, Edgington R, Bongrain A, Welch JO, Scorsone E, Bergonzo P, Jackman RB, Offenhäusser A (2014). Boron-doped nanocrystalline diamond microelectrode arrays monitor cardiac action potentials. Adv Healthc Mater.

[66] McDonald M, Monaco A, Vahidpour F, Haenen K, Giugliano M, Nesladek M (2017). Diamond microelectrode arrays for in vitro neuronal recordings. MRS Commun.

[67] Meunier A, Jouannot O, Fulcrand R, Fanget I, Bretou M, Karatekin E, Arbault S, Guille M, Darchen F, Lemaëtre F, Amatore C (2011). Coupling amperometry and total internal reflection fluorescence microscopy at ITO surfaces for monitoring exocytosis of single vesicles. Angew Chem Int Ed.

[68] Miccoli B, Lopez CM, Goikoetxea E, Putzeys J, Sekeri M, Krylychkina O, Chang SW, Firrincieli A, Andrei A, Reumers V, Braeken D (2019). High-density electrical recording and impedance imaging with a multi-modal CMOS multi-electrode array chip. Front Neurosci.

[69] Miller C (2009). Everything you always wanted to know about Sachs’ seals. Biophys J.

[70] Mohr M, Picollo F, Battiato A, Bernardi E, Forneris J, Tengattini A, Enrico E, Boarino L, Bosia F, Fecht HJ, Olivero P (2016). Characterization of the recovery of mechanical properties of ion-implanted diamond after thermal annealing. Diam Relat Mater.

[71] Mosca B, Delbono O, Laura Messi M, Bergamelli L, Wang ZM, Vukcevic M, Lopez R, Treves S, Nishi M, Takeshima H, Paolini C, Martini M, Rispoli G, Protasi F, Zorzato F (2013). Enhanced dihydropyridine receptor calcium channel activity restores muscle strength in JP45/CASQ1 double knockout mice. Nat Commun.

[72] Nistor PA, May PW, Tamagnini F, Randall AD, Caldwell MA (2015). Long-term culture of pluripotent stem-cell-derived human neurons on diamond – a substrate for neurodegeneration research and therapy. Biomaterials.

[73] Pages S, Cote D, De Koninck P (2011). Optophysiological approach to resolve neuronal action potentials with high spatial and temporal resolution in cultured neurons. Front Cell Neurosci.

[74] Penner R (1995) A practical guide to patch clamping. In: Sakmann B, and Neher E (ed) Single-channel recording, 2nd edn. Plenum Press, New York, pp 3-30

[75] Peterka DS, Takahashi H, Yuste R (2011). Imaging voltage in neurons. Neuron.

[76] Petrini G, Moreva E, Bernardi E, Traina P, Tomagra G, Carabelli V, Degiovanni IP, Genovese M (2020) Is a quantum biosensing revolution approaching? Perspectives in NV-assisted current and thermal biosensing in living cells. Adv Quantum Technol 2000066 10.1002/qute.202000066

[77] Picollo F, Battiato A, Bernardi E, Boarino L, Enrico E, Forneris J, Gatto Monticone D, Olivero P (2015). Realization of a diamond based high density multi electrode array by means of deep ion beam lithography. Nucl Instruments Mrethods Phys Res Sect B Beam Interact with Mater Atoms.

[78] Picollo F, Battiato A, Bernardi E, Marcantoni A, Pasquarelli A, Carbone E, Olivero P, Carabelli V (2016). Microelectrode arrays of diamond-insulated graphitic channels for real-time detection of exocytotic events from cultured chromaffin cells and slices of adrenal glands. Anal Chem.

[79] Picollo F, Battiato A, Bernardi E, Plaitano M, Franchino C, Gosso S, Pasquarelli A, Carbone E, Olivero P, Carabelli V (2016). All-carbon multi-electrode array for real-time in vitro measurements of oxidizable neurotransmitters. Sci Rep.

[80] Picollo F, Battiato A, Boarino L, Ditalia Tchernij S, Enrico E, Forneris J, Gilardino A, Jakšić M, Sardi F, Skukan N, Tengattini A, Olivero P, Re A, Vittone E (2017). Fabrication of monolithic microfluidic channels in diamond with ion beam lithography. Nucl Instruments Methods Phys Res Sect B Beam Interact with Mater Atoms.

[81] Picollo F, Gosso S, Vittone E, Pasquarelli A, Carbone E, Olivero P, Carabelli V (2013). A new diamond biosensor with integrated graphitic microchannels for detecting quantal exocytic events from chromaffin cells. Adv Mater.

[82] Picollo F, Olivero P, Bellotti F, Pastuović Ž, Skukan N, Lo Giudice A, Amato G, Jakšić M, Vittone E (2010). Formation of buried conductive micro-channels in single crystal diamond with MeV C and He implantation. Diam Relat Mater.

[83] Picollo F, Tomagra G, Bonino V, Carabelli V, Mino L, Olivero P, Pasquarelli A, Truccato M (2020). Triggering neurotransmitters secretion from single cells by X-ray nanobeam irradiation. Nano Lett.

[84] Piret G, Hébert C, Mazellier J-PP, Rousseau L, Scorsone E, Cottance M, Lissorgues G, Heuschkel MO, Picaud S, Bergonzo P, Yvert B (2015). 3D-nanostructured boron-doped diamond for microelectrode array neural interfacing. Biomaterials.

[85] Priel A, Gil Z, Moy VT, Magleby KL, Silberberg SD (2007). Ionic requirements for membrane-glass adhesion and giga seal formation in patch-clamp recording. Biophys J.

[86] Pusch M, Neher E (1988). Rates of diffusional exchange between small cells and a measuring patch pipette. Pflugers Arch.

[87] Qu D, Shi H (1998). Studies of activated carbons used in double-layer capacitors. J Power Sources.

[88] Reddy GD, Kelleher K, Fink R, Saggau P (2008). Three-dimensional random access multiphoton microscopy for functional imaging of neuronal activity. Nat Neurosci.

[89] Reyes AD (2019) A breakthrough method that became vital to neuroscience nature 575(7781):38-39. 10.1038/d41586-019-02836-6 10.1038/d41586-019-02836-6 31686045

[90] Richard A. Levis RA, Rae JL (2007) Technology of patch-clamp electrodes. In: Walz W (ed) Patch-clamp analysis: advanced techniques, 2nd edn. Series: Neuromethods (Book 38), Humana Press, Totowa, pp 1-34

[91] Rispoli G (1998). Calcium regulation of phototransduction in vertebrate rod outer segments. J Photochem Photobiol B.

[92] Rispoli G (2017). Studying the mechanism of membrane permeabilization induced by antimicrobial peptides using patch-clamp techniques. Methods Mol Biol.

[93] Rispoli G, Navangione A, Vellani V (1995). Transport of K^+^ by photoreceptor Na(^+^)-Ca^2+^,K^+^ exchanger in isolated rods of lizard retina. Biophys J.

[94] Roome CJ, Kuhn B (2014). Chronic cranial window with access port for repeated cellular manipulations, drug application, and electrophysiology. Front Cell Neurosci.

[95] Roome CJ, Kuhn B (2018). Simultaneous dendritic voltage and calcium imaging and somatic recording from Purkinje neurons in awake mice. Nat Commun.

[96] Roome CJ, Kuhn B (2019) Voltage imaging with ANNINE dyes and two-photon microscopy. In: Hartveit E (ed) Multiphoton microscopy. Springer Nature Neuromethods. Springer Nature (Neuromethods), pp 297-334. 10.1007/978-1-4939-9702-2_13

[97] Roome CJ, Kuhn B (2020). Voltage imaging with ANNINE dyes and two-photon microscopy of Purkinje dendrites in awake mice. Neurosci Res.

[98] Sartori AF, Belardinelli P, Dolleman RJ, Steeneken PG, Ghatkesar MK, Buijnsters JG (2019) Inkjet-printed high-Q nanocrystalline diamond resonators. Small 15. 10.1002/smll.201803774 10.1002/smll.201803774 30566284

[99] Schroeder TJ, Borges R, Finnegan JM, Pihel K, Amatore C, Wightman RM (1996). Temporally resolved, independent stages of individual exocytotic secretion events. Biophys J.

[100] Slavchov RI, Nomura T, Martinac B, Sokabe M, Sachs F (2014). Gigaseal mechanics: creep of the gigaseal under the action of pressure, adhesion, and voltage. J Phys Chem B.

[101] Slavík J, Skopalík J, Provazník I, Hubálek J (2019). Multi-electrode array with a planar surface for cell patterning by microprinting. Sensors (Basel).

[102] Suchyna TM, Markin VS, Sachs F (2009). Biophysics and structure of the patch and the gigaseal. Biophys J.

[103] Sulzer D, Cragg SJ, Rice ME (2016). Striatal dopamine neurotransmission: regulation of release and uptake. Basal Ganglia.

[104] Sun X, Gillis KD (2006). On-chip amperometric measurement of quantal catecholamine release using transparent indium tin oxide electrodes. Anal Chem.

[105] Suzuki I, Fukuda M, Shirakawa K, Jiko H, Gotoh M (2013). Carbon nanotube multi-electrode array chips for noninvasive real-time measurement of dopamine, action potentials, and postsynaptic potentials. Biosens Bioelectron.

[106] Theer P, Kuhn B, Keusters D, Denk W (2005) Two-photon microscopy and imaging. In: Meyers RA (ed) Encyclopedia of molecular biology and molecular medicine, vol 15. 2nd edn. VCH, Weinheim Germany; New York, pp 61-87. 10.1002/3527600906.mcb.200500019

[107] Tomagra G, Aprà P, Battiato A, Collà Ruvolo C, Pasquarelli A, Marcantoni A, Carbone E, Carabelli V, Olivero P, Picollo F (2019). Micro graphite-patterned diamond sensors: towards the simultaneous in vitro detection of molecular release and action potentials generation from excitable cells. Carbon N Y.

[108] Tomagra G, Franchino C, Pasquarelli A, Carbone E, Olivero P, Carabelli V, Picollo F (2019). Simultaneous multisite detection of quantal release from PC12 cells using micro graphitic-diamond multi electrode arrays. Biophys Chem.

[109] Tomagra G, Picollo F, Battiato A, Picconi B, De Marchis S, Pasquarelli A, Olivero P, Marcantoni A, Calabresi P, Carbone E, Carabelli V (2019) Quantal release of dopamine and action potential firing detected in midbrain neurons by multifunctional diamond-based microarrays. Front Neurosci 13:e288. 10.3389/fnins.2019.00288 10.3389/fnins.2019.00288 PMC6465646 31024230

[110] Tominaga T, Tominaga Y, Ichikawa M* (2001) Simultaneous multi-site recordings of neural activity with an inline multi-electrode array and optical measurement in rat hippocampal slices. Pflugers Arch 443:317-322. 10.1007/s004240100707 10.1007/s004240100707 11713660

[111] Van Kempen GTH, Vanderleest HT, Van Den Berg RJ, Eilers P, Westerink RHS (2011). Three distinct modes of exocytosis revealed by amperometry in neuroendocrine cells. Biophys J.

[112] Vanhove E, de Sanoit J, Mailley P, Pinault M-A, Jomard F, Bergonzo P (2009). High reactivity and stability of diamond electrodes: the influence of the B-doping concentration. Phys Status Solidi.

[113] Vedovato N, Rispoli G (2007). A novel technique to study pore-forming peptides in a natural membrane. Eur Biophys J.

[114] Wellman SM, Eles JR, Kip A, Seymour LJP, Michelson NJ, McFadden WE, Vazquez AL, Kozai TFY (2018). A materials roadmap to functional neural interface design. Adv Funct Mater.

[115] Wightman RM, Jankowski JA, Kennedy RT, Kawagoe KT, Schroeder TJ, Leszczyszyn DJ, Near JA, Diliberto EJ, Viveros OH (1991). Temporally resolved catecholamine spikes correspond to single vesicle release from individual chromaffin cells. Proc Natl Acad Sci U S A.

[116] Wong YT, Ahnood A, Maturana MI, Kentler W, Ganesan K, Grayden DB, Meffin H, Prawer S, Ibbotson MR, Burkitt AN (2018). Feasibility of nitrogen doped ultrananocrystalline diamond microelectrodes for electrophysiological recording from neural tissue. Front Bioeng Biotechnol.

[117] Wu JL, Liang YJ, Chen S, Hsu CL, Chavarha M, Evans SW, Shi DQ, Lin MZ, Tsia KK, Ji N (2020). Kilohertz two-photon fluorescence microscopy imaging of neural activity in vivo. Nat Methods.

[118] Yan P, Acker CD, Zhou WL, Lee P, Bollensdorff C, Negrean A, Lotti J, Sacconi L, Antic SD, Kohl P, Mansvelder HD, Pavone FS, Loew LM (2012). Palette of fluorinated voltage-sensitive hemicyanine dyes. Proc Natl Acad Sci U S A.

[119] Yang SY, Kim BN, Zakhidov AA, Taylor PG, Lee J-K, Ober CK, Lindau M, Malliaras GG (2011). Detection of transmitter release from single living cells using conducting polymer microelectrodes. Adv Mater.

[120] Zhang B, Adams KL, Luber SJ, Eves DJ, Heien ML, Ewing AG (2008). Spatially and temporally resolved single-cell exocytosis utilizing individually addressable carbon microelectrode arrays. Anal Chem.

[121] Ziegler JF, Ziegler MD, Biersack JP (2010). SRIM - The stopping and range of ions in matter (2010). Nucl Instruments Methods Phys Res Sect B Beam Interact with Mater Atoms.

